# Comparative transcriptome and coexpression network analysis reveals key pathways and hub candidate genes associated with sunflower (*Helianthus annuus L.*) drought tolerance

**DOI:** 10.1186/s12870-024-04932-w

**Published:** 2024-03-27

**Authors:** Huimin Shi, Jianhua Hou, Dandan Li, Haibo Hu, Yanxia Wang, Yang Wu, Liuxi Yi

**Affiliations:** College of Agriculture, Inner Mongolia Agricultural University, Hohhot, 010018 China

**Keywords:** Sunflower, Seedling drought, Transcriptome, GO and KEGG, Transcription factor, WGCNA, Homology alignment

## Abstract

**Background:**

Drought severely limits sunflower production especially at the seedling stage. To investigate the response mechanism of sunflowers to drought stress, we utilized two genotypes of sunflower materials with different drought resistances as test materials. The physiological responses were investigated under well-watered (0 h) and drought-stressed conditions (24 h, 48 h, and 72 h).

**Results:**

ANOVA revealed the greatest differences in physiological indices between 72 h of drought stress and 0 h of drought stress. Transcriptome analysis was performed after 72 h of drought stress. At 0 h, there were 7482 and 5627 differentially expressed genes (DEGs) in the leaves of K55 and K58, respectively, and 2150 and 2527 DEGs in the roots of K55 and K58, respectively. A total of 870 transcription factors (TFs) were identified among theDEGs, among which the high-abundance TF families included AP2/ERF, MYB, bHLH,and WRKY. Five modules were screened using weighted gene coexpressionnetwork analysis (WGCNA), three and two of which were positively and negatively, respectively, related to physiological traits. KEGG analysis revealedthat under drought stress, “photosynthesis”, “carotenoid biosynthesis”, “starch and sucrose metabolism”, “ribosome”, “carotenoid biosynthesis”, “starch and sucrose metabolism”, “protein phosphorylation” and “phytohormone signaling” are six important metabolic pathways involved in the response of sunflower to drought stress. Cytoscape software was used to visualize the three key modules, and the hub genes were screened. Finally, a total of 99 important candidate genes that may be associated with the drought response in sunflower plants were obtained, and the homology of these genes was compared with that in *Arabidopsis thaliana*.

**Conclusions:**

Taken together, our findings could lead to a better understanding of drought tolerance in sunflowers and facilitate the selection of drought-tolerant sunflower varieties.

**Supplementary Information:**

The online version contains supplementary material available at 10.1186/s12870-024-04932-w.

## Introduction

Sunflower (*Helianthus annuus L.*) is an herbaceous plant in the Asteraceae family and is the third most important oilseed crop in the world. For a long time, sunflower has been regarded as a crop with salinity tolerance, barrenness tolerance, drought resistance, and high adaptability. However, drought is a frequent problem in the main sunflower-producing areas such as Inner Mongolia and Xinjiang, often due to insufficient precipitation and uneven spatial and temporal distributions, and spring droughts are particularly serious. This results in an insufficient water supply for seed germination and seedling growth, leading to the slow emergence of sunflower seedlings, poor root development, thin and weak seedlings, reduced plant height, reduced leaf area, a shortened fertility period, and, ultimately, significantly lower yield quality [1, 2]. In addition, although sunflower has a well-developed root system for efficient water extraction, leaf transpiration rates can reach very high values [3], thus exacerbating the risk of drought stress in sunflowers. It has been shown that drought-induced yield losses in sunflowers each approximately 50% [4]. Therefore, it is of great practical significance to study the mechanism of drought resistance in sunflower and to understand its physiological and molecular responses to drought stress to accelerate the selection of new drought-resistant (tolerant) varieties and to ensure high and stable yields of sunflower.

Plants sense stress signals and undergo transcriptional reprogramming to induce physiological and biochemical changes under drought stress [5]. Antioxidant enzymes help eliminate excess ROS accumulationunder abiotic stress conditions to prevent oxidative damage in plants [6]. In addition, soluble sugars and proline (Pro)act as osmotic substances to protect plants fromstress. In addition to the total antioxidant capacity and osmotic regulation, the concentrations of malondialdehyde (MDA) and plant hormones are important indicators of drought resistance in plants. Specifically, the MDA content is used as a marker of oxidative lipid damage to reflect the response of plants to stress [7, 8]. As an important signalling molecule, plant hormones resist or adapt to drought stress by jointly responding to water deficit under drought stress. In particular, ABA, a typical drought signalling substance, functions through signal transduction [9].

Furthermore, plants have evolved a complex response mechanism for survival and growth under drought stress, which involves the expression of a range of related genes and posttranscriptional regulators [10]. There are two main types of drought resistance genes. One is a functional gene whose coding product plays a direct protective role in plant drought resistance. Othertranscriptional regulators regulate the expression of genes associated with adversity, such as *AP2*/*ERF*, *bHLH*, *bZIP*, *GATA*, *HD-Zip*, *MYB*, *NAC*, *WHIRLY*, *WOX*, *WRKY*, and zinc fingers. These genes function in both ABA-dependent and ABA-independent signalling pathways [11, 12]. Only *HaWRKY76* [13], *HaHB4* [14], and *HaHB11* [15] have been reported to be involved in the regulation of the drought stress response in sunflowers through transgenic technology, and additional genes have not been functionally validated. Therefore, the isolation and characterization of drought-related genes are important for elucidating the molecular mechanisms of drought resistance in sunflower and for breeding drought-resistant sunflower varieties.

It is well known that the development of drought-tolerant sunflower varieties is an effective way to overcome water limitations under drought conditions. The isolation and identification of drought tolerance-related genes are important for revealing the molecular mechanism of drought tolerance in sunflowers and for breeding drought tolerant sunflowervarieties [16]. In plants, a number of candidate genes or loci have been identified using quantitative trait locus (QTL) positioning and genome-wide association analysis (GWAS) [17–20]. However, there are still some challenges in the use of these approaches to accurately identify drought resistance genes. Fortunately, with the publication of the sunflower genome sequence [21] and the development of next-generation sequencing technologies, transcriptome analysis provides a powerful tool to gain insights into the molecular mechanisms of sunflower responses to drought stress. RNA-Seq allows for fully quantitative gene expression analysis with absolute values and the capture of very subtle expression changes. Moreover, this technique provides low-cost, high-throughput, and high-sensitivity analysis of data [22, 23]. RNA-seq technology has been employed in drought stress response studies in sunflowers. S Moschen et al. [24] examined transcriptomics and metabolomics in three developmental stages (seedling,preflowering and postflowering) of sunflower leaves after drought stress. Through comprehensive analysis, they found that some transcription factors whose expression was upregulatedunder drought conditions were not only related to the drought responsebut also might be hubs in the transcriptional network. Liang et al. [25] reported the tissue specificity of gene expression in response to drought stress in sunflower seedlings after 24 h of PEG stress. In addition, there were more differentially expressed genes (DEGs) in the leaves than in the roots. Wu et al. [26] performed transcriptome sequencing of sunflower seedlings under different drought stress time points and screened several functional genes and transcription factors closely related to drought stress. This study provides new insights into the drought response mechanism of sunflowers.

However, all of the above studies used only one genotype or one sunflower tissue and did not analyse both different tissues and different genotypes. According to Sarazin Vivien et al. [23] and Liang et al. [24], there are not only genotypic differences but also tissue specificity in plant responses to water stress. Therefore, in this study, we sampled sunflower leaves and roots under drought stress using the sunflower inbred lines K55 and K58. At the same time, we combined comparative transcriptomics and coexpression network analysis to screen the major regulatory genes involved in the response of sunflowers to drought stress and preliminarily elucidated the molecular regulatory mechanism of drought tolerance in sunflowers, which provides a theoretical basis for breeding new drought-tolerant sunflower varieties. More importantly, the cloning and expression analysis of DEGs identified in this study can provide multiple candidate genes and information for improving the drought resistance of sunflower plants via genetic engineering technology.

## Materials and methods

### Plant materials and growth conditions

The drought-tolerant variety K58 (abbreviation K) and drought-sensitive variety K55 (abbreviation B), which were selected from the 226 varieties in our previous study were used to evaluate drought tolerance [27]. The research was conducted in the city of Hohhot (111.71, 40.819, and 1000 m above sea level), Inner Mongolia Province, China). in early October 2021). Seeds were sterilized with 3% sodium hypochlorite for 5 min, followed by washing 3 times with sterile distilled water. Then, the sterilized seeds were placed in sterile Petri dishes pre-positioned with two layers of wet filter paper and germinated at room temperature (19°C to 25°C) for 24 h. The germinated seeds were sown in polyvinyl chloride pots (25 cm × 19 cm × 16 cm) pre-filled with 4 kg of soil (75% sand:20% nutrient soil:5% vermiculite) and planted with six seeds in each pot in a greenhouse (light/dark cycle:14h/10h; 25°C; 45 ± 5% relative humidity) without water or nutritional limitations.

### Drought treatments

According to Pereyra et al. [28], irrigation was stopped for all seedlings after two pairs of leaves fully expanded. Every day at 8 a.m., the soil water content was monitored using a soil water content meter (TZS, TOP Instruments, China), and the water was replenished according to the target soil water content. We controlled the soil water content under drought onditions to 30% and recorded the soil water content at 8 a.m. on the day when the soil water content first decreased to 30% at 0 h. Seedlings of both varieties were subjected to drought stress for 0, 24, 48, and 72 h. Subsequently, samples were taken to determine physiological indices after drought treatments of 0, 24, 48, and 72 h, and each treatment was replicated three times. Fresh tissue was used to determine physiological indices. To eliminate experimental errors, the same physiological indices were measured at the same leaf and root positions. Moreover, samples for transcriptome assays were collected, immediately frozen in liquid nitrogen, and stored at -80 °C until use. To distinguish between leaves and roots, we used “L” for leaves and “R” for roots.

### Determination of physiological indices

All physiological indices, namely, superoxide dismutase (SOD) activity, malondialdehyde (MDA) content, Proline content, and ABA content, were determined according to Plant SOD ELISA Kit, Plant MDA ELISA Kit, Plant proline ELISA Kit and Plant ABA ELISA Kit (Shanghai Preferred Bio). Enzyme-linked immunosorbent (ELISA) assay. For different treatments, sunflower leaves and roots were ground into powder in liquid nitrogen, and 0.1 g of each sample was accurately weighed in a centrifuge tube. Subsequently, an equal volume of 0.1 mol/L precooled PBS solution was added, and the mixture was centrifuged at 1000 × g for 20 min. The supernatant was collected and assayed according to the manufacturer’s instructions [29], and the absorbance as the optical density (OD) was measured at 450 nm with a microplate ELISA reader (Zenyth 3100 ELISA reader). The linear regression curves of each indicator standard were plotted by taking the concentration of each indicator standard as the horizontal coordinate and the corresponding OD value as the vertical coordinate, and the content of each sample was calculated according to the curve equation. The data were analysed by one-way analysis of variance (ANOVA) using SPSS statistical software (SPSS version 20.0). Bar graphs were generated with GraphPad Prism (v8.0.2).

### RNA extraction, transcriptome sequencing, and data analysis

By analysing physiological traits, we screened the two materials with the greatest differences between 0 and 72 h of drought stress. Therefore, a transcriptome assay was performed using the leaves and roots of sunflower seedlings after 0 h and 72 h of drought stress. Three replicates were taken for each treatment, and RNA was extracted. RNA was extracted and tested for quality, concentration, and integrity according to Wu et al. [26]. After the samples were tested, library construction was carried out, and the main processes were as follows: (1) eukaryotic mRNA was enriched with magnetic beads with Oligo (dT); (2) mRNA was randomly interrupted by the addition of fragmentation buffer; (3) mRNA was used as a template to synthesize the first cDNA strand, and then the second cDNA strand was synthesized by adding buffer, dNTPs, RNase H and DNA polymerase I. The cDNA was purified by using AMPure XP beads; (4) the purified double-stranded cDNA was then subjected to end repair, the addition of A-tail and ligation to sequencing junctions, and then fragment size selection was performed by using AMPure XP beads; (5) finally, the cDNA library was enriched by PCR.

After library construction was completed, the effective concentration of the libraries (> 2nM) was accurately quantified by Q-PCR to ensure the quality of the libraries. After the libraries were qualified, different libraries were pooled according to the target downstream data volume, and sequenced on the Illumina platform, which was completed by Beijing Biomarker Technology Company. After filtering the raw reads, high-quality clean reads were obtained with the latest version of the sunflower reference genome (GenBank NO. GCA_002127325.2) [21] using HISAT2 (version 2.2.1) software [30] with default parameters.

The SAM files were converted to BAM files and sorted using SAMtools software. StringTie (software v.2.2.0) was used to generate gtf files for each material. The gtf files were merged using the ‘-merge’ parameter. The ‘prepDE.py3’ python package was used to obtain the gene count [31]. The DEGs with FPKM values > 0 were subjected to correlation heatmap and principal component analysis using the Corrplot R package. DEGs were analysed using DESeq2 (3.6.3) software [32]. These samples were divided into four groups (K55L0h vs. K55L72h, K55R0h vs. K55R72h, K58L0h vs. K58L72h, K58R0h vs. K58R72h). Genes with padj ≤ 0.01 and |log2(fold changes) |≥ 1 were considered DEGs. *p*-values were adjusted using the Benjamini and Hochberg method. The Fasta file was imported into Eggnog (v 2.0.1) [33] for functional annotation. Based on the annotation of gene function, we performed enriched GO and KEGG analyses of DEGs under different combinations of differences. The REVIGO program (http://revigo.irb.hr/) was used to remove redundant GO terms [34]. MapMan software was used to visualize plant metabolic pathways and gene expression. Gene annotations were obtained using Mercator software. Briefly, DNA sequences were extracted from the genome using gffread software. The obtained sequences were uploaded and genetically annotated using Mercator software with default parameters (https://www.plabipd.de/mercator_main.html). Transcription factors were identified and classified by uploading gene sequences to the iTAK website [35].

### Weighted gene coexpression network analysis

The WGCNA R package was used to construct a coexpression network of all 12,892 DEGs. [36]. Poorly representative samples were removed using the stereotree static function. The β value was determined based on the scale-free topological fit index (*R*^2^ > 0.85) and low average connectivity. The Cytoscape function was used to output network edge and node information for genes in each module. GO term and KEGG pathway enrichment analyses were performed on the genes in each module to reveal the biological functions of the modules. The REVIGO program (http://revigo.irb.hr/) was used to remove redundant GO terms, and heatmaps were generated to visualize nonredundant GO terms. Cytoscape (version 3.9.1) [37] was used to visualize the networks. We used the cytoHubba plugin (http://apps.cytoscape.org/apps/cytohubba) to identify the top 50 hub genes via maximum clustered centrality (MCC) calculations.

### RNA-seq data validation of DEGs

Quantitative real-time PCR (RT‒qPCR) was performed to validate the differentially expressed loci obtained from the RNA-seq data. Therefore, we randomly selected 10 DEGs for RT‒qPCR analysis. Total RNA was extracted from 24 samples with TRIzol reagent (Sheng Gong, Beijing, China). Reverse transcription was performed using standard procedures of the Biomarker Script II1st Strand cDNA Synthesis Kit (Biomarker). RT‒qPCR analysis was performed in an Applied Biosystems TM QuantStudioTM3&5 real-time quantitative PCR instrument (Thermo Fisher Scientific, Shanghai, China) using KWBIO 2MgicSYBRMixture (Jiangsu, China). All gene-specific primers were designed using Primer Premier 6 softwareand synthesized by Sangon Biotech Co., Ltd. The details of the primers used are shown in Supplementary Table 1. The Ha18S rRNA gene (*LOC118483140*) was used as an internal reference gene [38]. The relative expression of genes was analysed by the 2^−∆∆Ct^ method [39]. The verification experiment was performed in three biological replicates, with three technical replicates in each replicate. The resultant plot was produced by Origin 2022.

### Screening of candidate genes and homology comparison

Candidate genes were screened by GO, KEGG, transcription factor prediction, and WGCNA with FPKM values, and then the protein sequences of the candidate genes were searched on NCBI. The candidate genes were homologous to the genome of Arabidopsis 11 on the TAIR website (https://www.arabidopsis.org).

## Results

### Physiological responses to drought stress in sunflowers

**Fig. 1:**
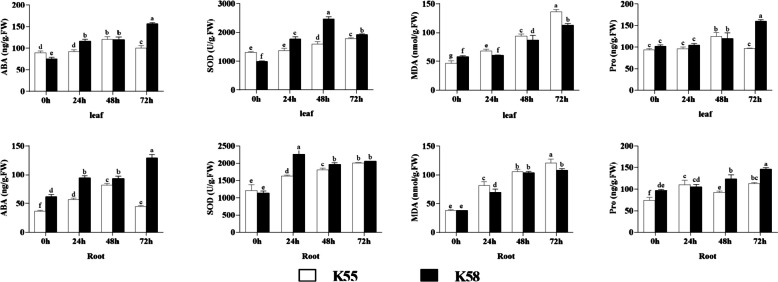
Physiological and biochemical traits of K55 and K58 under drought stress. Error bars denote standard error of the mean. Significant differences between samples at *p* <  = 0. 05 were denoted by different letters

To understand the different response mechanisms to drought stress in sunflowers, we investigated the physiological and biochemical changes in the leaves and roots of plants of both genotypes under different stress durations (Fig. 1). Notably, the ABA content in the leaves and roots of both materials increased after drought stress. However, the ABA content of K55 peaked at 48 h and decreased with increasingdrought stress. However, the ABA content of K58 increased throughout the drought stress period and reached a maximum at 72 h. The ABA content of K55 reached a maximum at 48 h and decreased with increasing drought stress. The ABA content in K58 increased at 48 h and decreased with increasing drought stress duration. Superoxide dismutase (SOD) activity increased in both materials after drought stress, with K55 showing a continuous increase. However, changes in activity in K58 leaves and roots were different. The SOD activity in leaves reached the maximum value after 48 h and then decreased, whereas the SOD activity in roots reached the maximum value after 24 h and then decreased. In addition, the SOD activity in K58 was greater than that in K55 except at 0 h. Drought stress resulted in a sustained increase in the MDA content, which was greaterr in K55 than in K58. The Pro content also increased after drought stress. Importantly, the Pro content of K55 leaves and roots reached a maximum at 48 and 24 h, respectively, and was greater than that of K58 at both time points. In K58, the Pro content increased continuously from the beginning of drought stress to 72 h, reached a maximum at 72 h and was significantly greaterthan that in K55. Through ANOVA, we found the most significant differences in ABA, MDA, and Pro contents at 0 h and 72 h compared to those in the other periods for both materials, except for SOD. The differences in the physiological responses of leaves and roots to drought stress between the two materials suggest that there is tissue specificity in the response to drought stress in the different materials.

### Transcriptome sequencing and mapping

To reveal the molecular mechanisms underlying the differences in drought tolerance between K55 and K58 at the seedling stage, we performed transcriptome sequencing of the leaf and root tissues with the most significant differences. Twenty-four cDNA libraries were constructed and sequenced by Beijing Biomarker Biotechnology Co., and a total of 155.26 Gb of clean data were obtained after quality control. Each library generated approximately 5.72 Gb of clean data. In addition, all the RNA-seq raw datasets were stored in the NCBI database under theSRA accession number PRJNA1041959. After removing reads containing adapters and low-quality reads, the Q30 of each library ranged from 92.81% to 94.47%, and the GC content ranged from 44.03% to 45.68%. Approximately 86.56% to 91.76% of clean reads in each library were mapped to the sunflower genome (https://www.ncbi.nlm.nih.gov/genome/annotation_euk/Helianthus_annuus/101/), of which 82.62% to 87.02% were uniquely mapped. Details of transcriptome sequencing and alignment with the reference genome are shown in Supplementary Table 2.

**Fig. 2:**
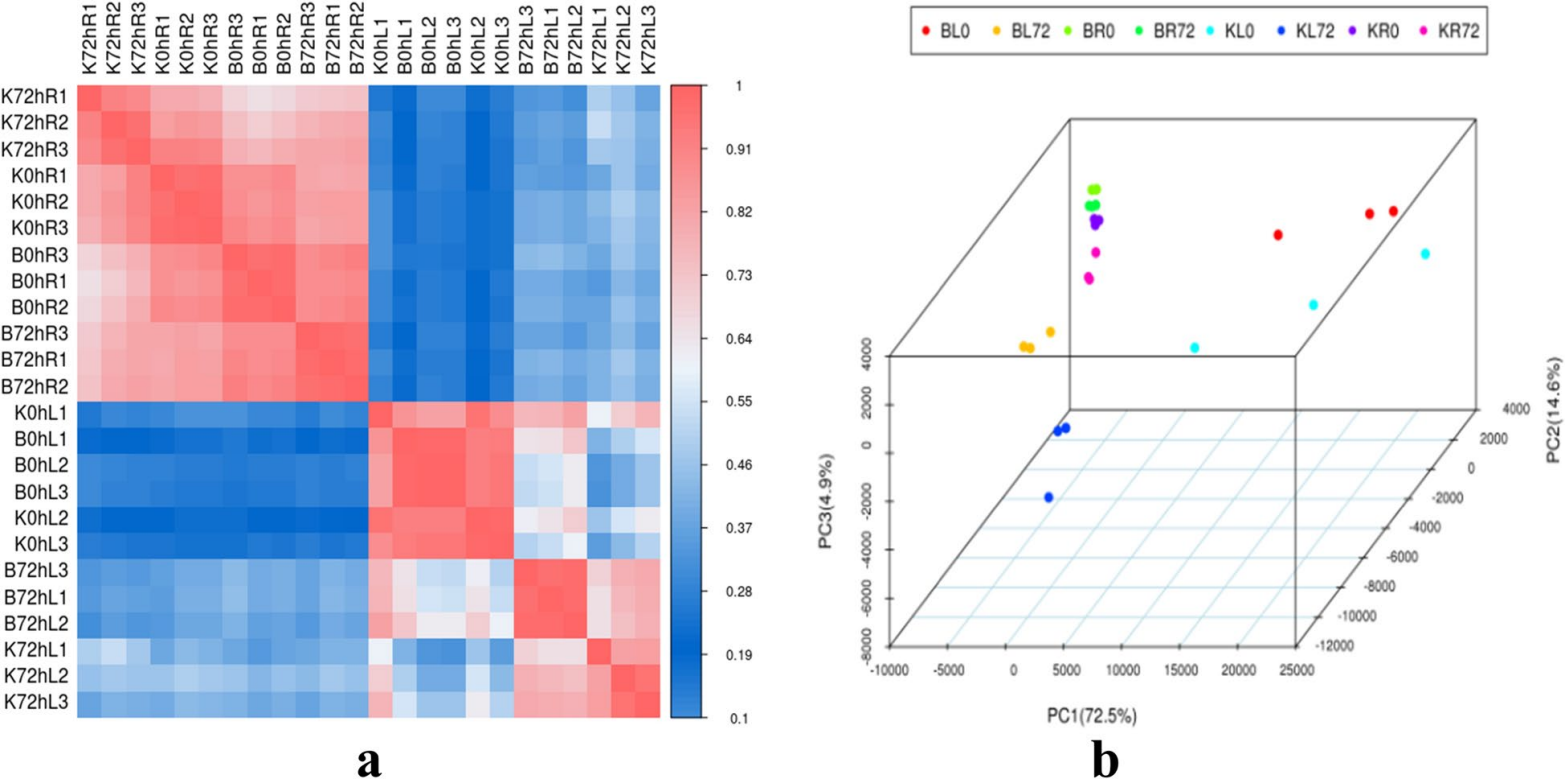
Transcriptional relationship among twenty-four samples. **a** Correlation coefficient analysis. **b** Principal component analysis (PCA)

Pearson’s correlation coefficient analysis based on the expression levels of all expressed genes revealed that the correlation coefficients between biological replicates ranged from 0.89 to 1.00. The above results indicate that the reproducibility between samples is reliable and our samples are representative (Fig. 2a).

PCA of the transcript expression of both genotypes revealed that the samples were closely clustered among the three replicates except for KL0, which was consistent with the results of the correlation analysis. In addition, the two genotypes were found to cluster separately under the two stress time points, indicating that the two genotypes had significantly different response mechanisms to drought stress. Leaf and root samples of the same material were also clustered separately, indicating that different sunflower tissues have different response mechanisms to drought stress (Fig. 2b).

### Differential expression gene analysis

**Fig. 3:**
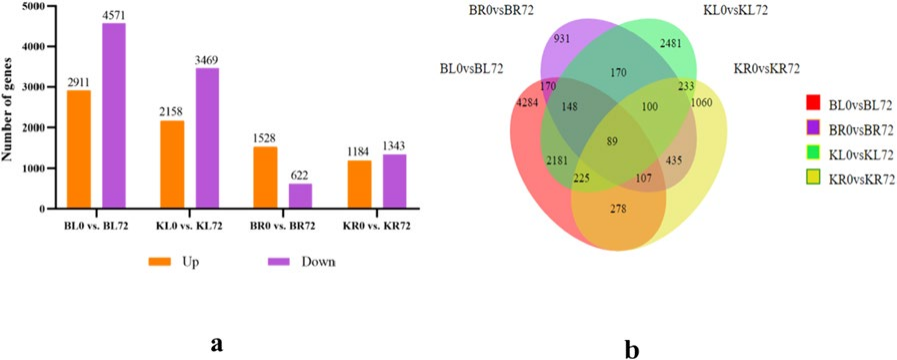
Statistics on the number of differentially expressed genes in each differential combination under drought stress and Venn diagram between differential groups. **a** Statistics on the number of differentially expressed genes. **b** Venn diagram analysis of expressed genes between differential groups

A total of 12,892 DEGs were obtained at two drought stress time points for the two varieties. There were 7482 and 5627 DEGs in the leaves of K55 and K58, respectively, with more upregulated and downregulated genes in K55 than in K58. In addition, 4284 DEGs were expressed only in K55 leaves, while 2481 DEGs were expressed less in K58 leaves than in K55 leaves, indicating that drought stress stimulated more gene expression in K55 leaves. In the roots, 2150 and 2527 DEGs were found in K55 and K58, respectively, with more upregulated genes in K55 than in K58 and fewer downregulated genes in K58 (Fig. 3a). A total of 931 of these DEGs were expressed only in K55 roots, and 1060 were expressed only in K58 roots. This indicates that in roots, the response of K58 to drought stress is more complex than that of K55. Overall, more DEGs were found in the leaves than in the roots of both genotypes under drought stress. This suggests that these genes may be regulated in a tissue-specific manner and thus may play a role in different mechanisms in response to drought stress. Interestingly, 170 and 233 DEGs were expressed simultaneously in the leaves and roots of K55 and K58, respectively (Fig. 3b). This indicates that leaves and roots overlap at the transcriptional level under drought stress.

### Analysis of common GOs enriched in two sunflower materials under drought stress

To reveal the functional differences of DEGs, GO enrichment analysis was carried out using all DEGs in the leaves and roots of the two materials (*P* value < 0.05). The GO terms were subdivided into three categories: biological process (BP), molecular function (MF), and cellular component (CC) (Supplementary Fig. 2). To further understand the differences in biological function between genotypes and between tissues, we compared the genes contained in the most important BP-like GO terms between different tissues of the same material and between the same tissues of different materials.

**Fig. 4:**
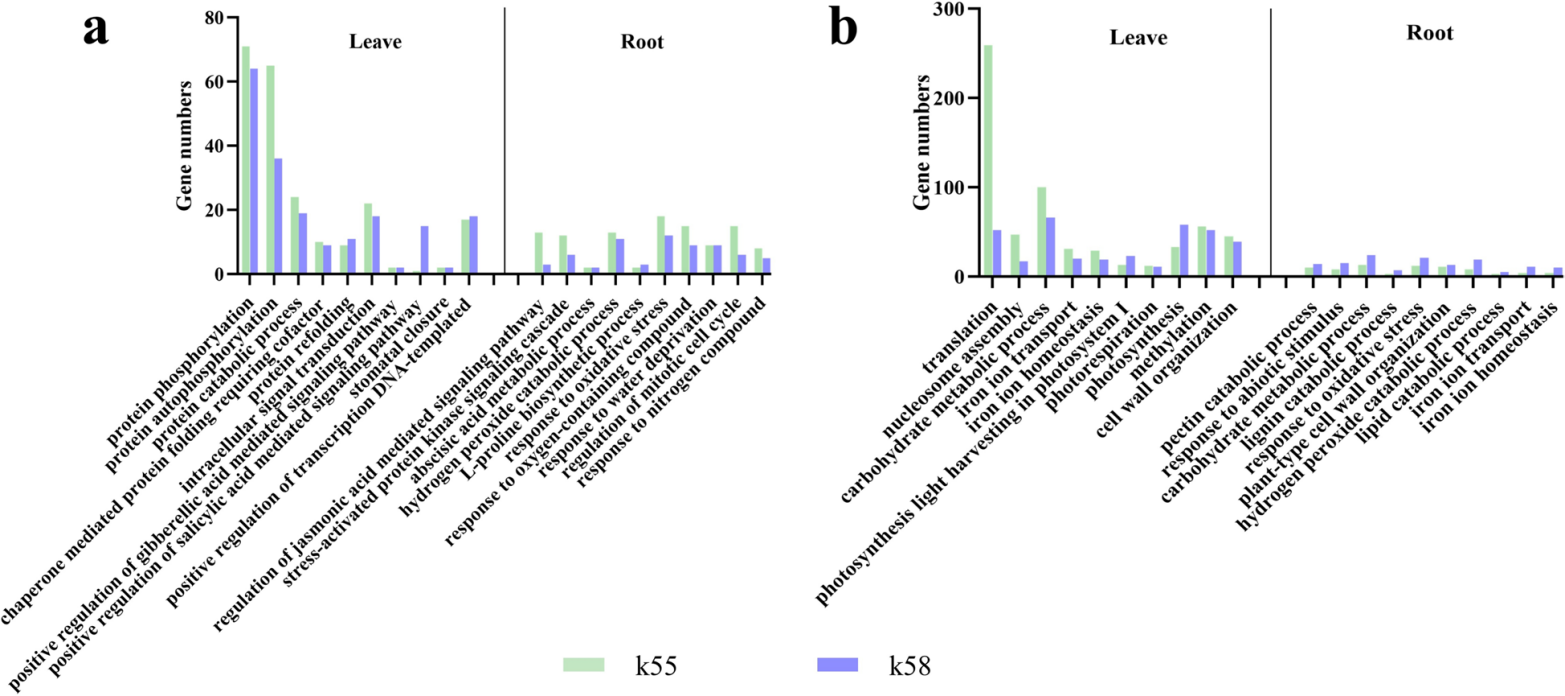
Common Go-term plots for leaves and roots of both materials, respectively. **a** up-regulated DEGs. **b** down-regulated DEGs

For the upregulated genes, 41 and 37 common GO terms were enriched in the leaves and roots, respectively. The DEGs in the leaves were mainly enriched in protein phosphorylation, catabolism, folding (GO: 0006468, GO: 0046777, GO: 003030163, GO: 0050821, GO: 0042026), and intracellular signalling (GO: 0035556, GO: 0009939, GO: 0080151). The DEGs in roots were mainly enriched in signalling pathways and signalling cascades (GO: 2000022, GO: 0031098), abiotic stress response (GO: 0009611, GO: 0010200, GO: 0006979, GO: 1901700, GO: 0009414, GO: 0042221, GO: 0009644, GO. GO: 1901698, GO: 0009631, GO: 0050896, GO: 0006950) and hormonal metabolic processes (GO: 0009687, GO: 0045487, GO: 0055129). In both leaves and roots, the number of DEGs enriched for common GO terms was greater in K55 than in K58 (Fig. 4a).

Among the downregulated genes, 48 and 23 common GO terms were enriched in the leaves and roots, respectively. DEGs in leaves were mainly enriched in translation (GO: 00064142, GO: 0032544, GO: 0006414, GO: 00510183), photosynthesis (GO: 0009768, GO: 00098, GO: 0009853, GO: 0010196, GO: 0015979) and carbon metabolism (GO: 0005975, GO: 0006730). DEGs in the roots were mainly enriched in the catabolic processes of organic matter (GO: 0045490, GO: 0031222, GO: 0005975, and GO: 0046274) and lipid biosynthesis and metabolism (GO: 0046471, GO: 0006636, GO: 0046506, GO: 0016042, and GO: 0046856). Interestingly, the downregulated genes were enriched for iron ion transport and iron ion homeostasis in the leaves and roots of both genotypes. In leaves, the number of DEGs in common GO terms enriched in K55 was greater than that in K58, except for photosynthesis-related pathways. But the opposite trend wasobserved for the roots (Fig. 4b).

### Analysis of common KEGG pathways enriched in the two sunflower materials under drought stress

KEGG enrichment analysis was subsequently conducted to identify pathways associated with these DEGs. We compared the differences in the number of genes enriched in the common KEGG pathways between the two gene types of leaves and roots (Supplementary Fig. 3). In addition, 4269 and 1014 DEGs were assigned to 255 and 180 KEGG pathways, respectively, in the leaves and roots of K55 under drought conditions. In addition, 3389 and 1246 DEGs were assigned to 250 and 216 KEGG pathways, respectively, in the leaves and roots of K58 under drought conditions. Subsequently, we selected pathways with a P value < 0.05 for the comparative study.

Overall, the number of downregulated genes was greater than the number of upregulated genes in all other combinations of differences except for the difference between K55R0 and K55R72. Thus, there were fewer common pathways enriched for upregulated genes than for downregulated genes in both genotypic materials.

**Fig. 5:**
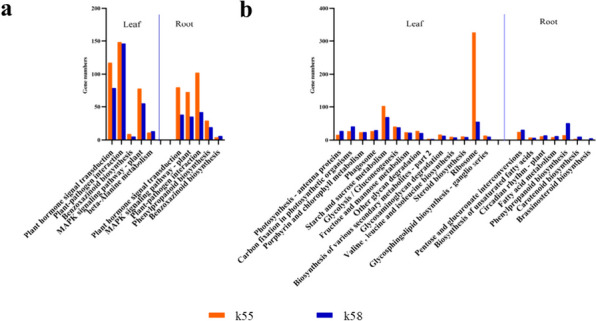
Common KEGG for leaves and roots of both materials, respectively. **a** up-regulated DEGs. **b** down-regulated DEGs

For upregulated DEGs, Plant hormone signal transduction (ko04075), MAPK signalling pathway—plant (ko04016), plant‒pathogen interaction (ko04626), and benzoxazinoid biosynthesis (ko00402) were enriched in the leaves and roots of both genotypes. In addition, the number of genes in K55 was greater than that in K58 in all common pathways except benzoxazinoid biosynthesis (ko00402) (Fig. 5a).

For downregulated DEGs, for both genotypes, the common pathways enriched in leaves and roots were completely different. A total of 14 pathways were enriched in the leaves, while only 7 pathways were coenriched in the roots. In the leaves, more photosynthesis-related pathways and sugar metabolism-related pathways, such as photosynthesis—antenna proteins (ko00196), carbon fixation in photosynthetic organisms (ko00710), and starch and sucrose metabolism (ko00500), were identified. However, the top pathways in the roots were phenylpropanoid biosynthesis (ko00940), pentose and glucuronate interconversions (ko00040) and biosynthesis of unsaturated fatty acids (ko01040) (Fig. 5b).

### Transcription factor prediction

**Fig. 6:**
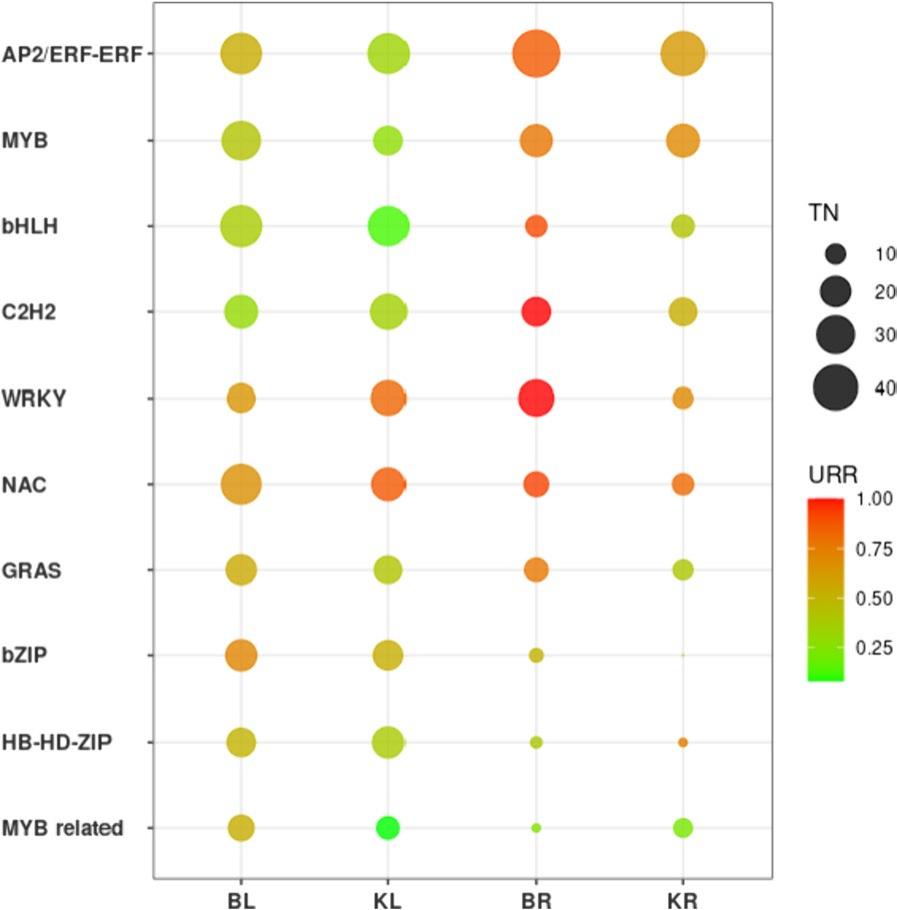
Ten transcription factor families with the largest number of genes. The size of the circle represents the total number of transcription factors, and color represents the proportion of up/downrelated genes in total genes, The redder the color, the more genes are up-regulated, and the greener the more genes are down-regulated

In this study, a total of 870 transcription factors(TFs), 723 protein kinases(PKs), and 133(TRs) were identified from all 12,892 DEGs. The top 10 TF families with the highest abundance were *AP2*/*ERF-ERF* (98 genes), *MYB* (870 genes), *bHLH* (62 genes), *C2H2* (55 genes), *WRKY* (55 genes), *NAC* (52 genes), *GRAS* (40 genes), *bZIP* (36 genes), *HB-HD-ZIP* (31 genes) and *MYB*-related (26 genes) (Fig. 6).

We analysed the expression of each transcription factor family in leaves and roots in both plant lines. Most of the transcription factor families, except the *AP2*/*ERF-ERF* family, were more highly expressed in K55 and K58 leaves than in roots.

In the leaves, the expression of the *MYB*, *bHLH*, and *MYB*-related families of transcription factors decreasedin K58, with upregulation rates of 27.77%, 12.5%, and 8.33%, respectively. *WRKY*, *NAC*, and *bZIP* families were highly upregulated in both materials. WRKY and NAC were more highly upregulated in K58 than in K55 (83.33% and 86.95%, respectively), while the opposite was true for *bZIP*.

In the root system, the *MYB*-related family of transcription factors had the lowest upregulation rates in K55 and K58 compared to the other transcription factor families, 25% and 22.2%, respectively. In contrast, transcription factors belonging to the *AP2*/*ERF-ERF*, *MYB*, *bHLH*, *C2H2*, *WRKY*, *NAC*, and *GRAS* families exhibited relatively high upregulation rates. Unlike in leaves, all transcription factor families except the *HB-HD-ZIP* family had higher upregulation rates in K55 than in K58. The upregulation rates in K55 were 86.66%, 77.27%, 90.9%, 100%, 100%, 92.85%, and 76.92%, respectively.

**Fig. 7:**
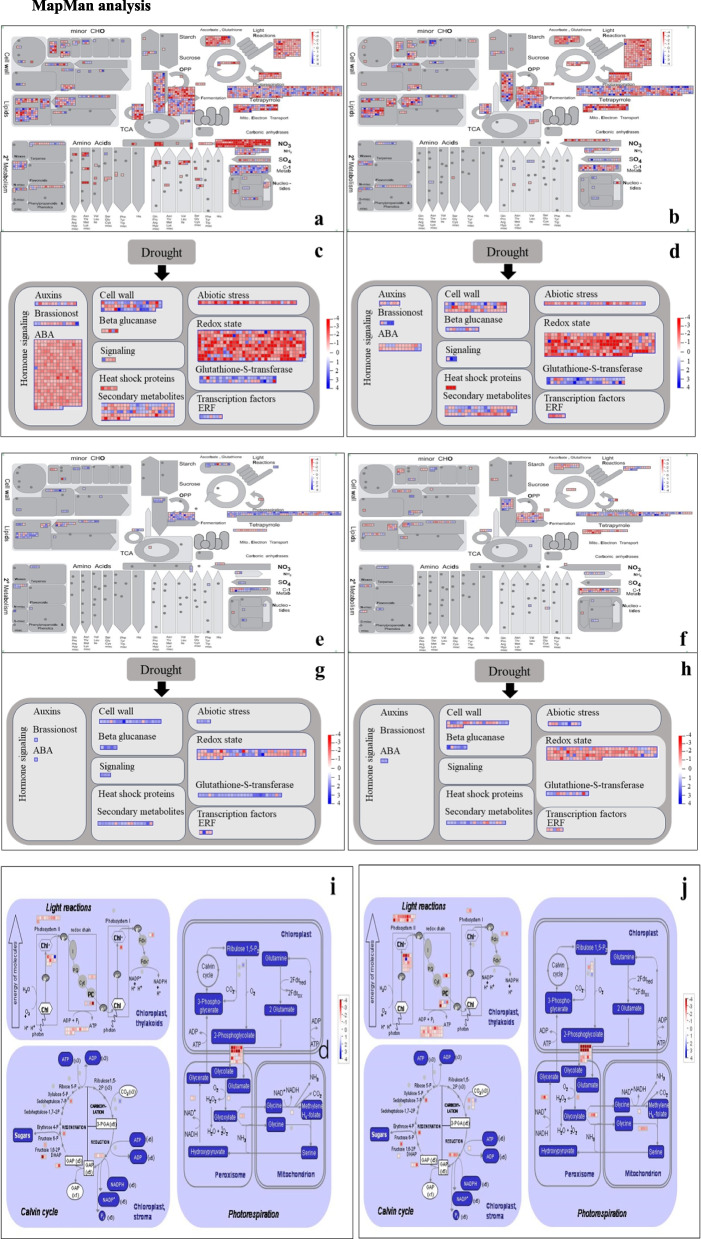
MapMan visualisation of DEGs in response to drought stress in leaves and roots of two genotypes under drought stress. Note: Each square represents a gene, blue indicates upregulationand red indicates downregulation. **a** Metabolic pathway profiles of K55 leaves at 72h of drought stress. **b** Metabolic pathway profile of K58 leaves at 72h of drought stress. **c** Metabolic pathways of K55 leaves at 72h of drought stress in drought stress. **d** Drought stress metabolic pathways of K58 leaves at 72h of drought stress. **e** Metabolic pathway profile of K55 roots at 72h of drought stress. **f** Metabolic pathway profile of K58 root system at 72h of drought stress. **g** Metabolic pathway of drought stress in K55 root system at 72h of drought stress. **h** Drought stress metabolic pathways in K58 root system at 72h of drought stress. **i** Photosynthetic metabolic pathway of K55 leaves at 72h of drought stress. **j** Photosynthetic metabolic pathway of K58 leaves at 72h of drought stress

The metabolic pathways of DEGs in the leaves and roots of the two species were analysed using the visualization software MapMan. The overall metabolism of K55 and K58, the different expression patterns of related genes under abiotic stress, and the differences in the expression of related genes in the leaf photosynthetic pathway are illustrated in Fig. 7.

From the overall metabolic profile, the metabolism and transcription factors of “cell wall”, "terpenes", and "starch and sucrose" were basically the same as those analysed by GO and KEGG. In addition, the number of genes enriched in each metabolic pathway was significantly lower in the roots than in the leaves for both materials. This suggests that leaves are more sensitive to drought stress and that the regulatory mechanisms involved are more complex. Because of the important roles of phytohormones, redox reactions, and photosynthesis in abiotic stress, we focused on these pathways. We found that only three hormones, auxin, brassinosteroid, and ABA, were enriched under drought stress, and the number of genes enriched in K55 was greater than that in K58. In particular, the difference in the number of ABA-related genes enriched in leaves was highly significant, with 23.08% of the genes upregulated in K58, compared with only 0.01% in K55. In addition, the number of genes enriched in each metabolic pathway was significantly lower in the roots than in the leaves. This suggests that leaves are more sensitive to drought stress than roots and that the regulatory mechanisms involved are more complex. The photosynthesis pathway was enriched with more genes in K58 leaves than in K55 leaves, but the proportion of downregulated genes was greater (Fig. 7).

### Weighted gene coexpression network analysis

**Fig. 8:**
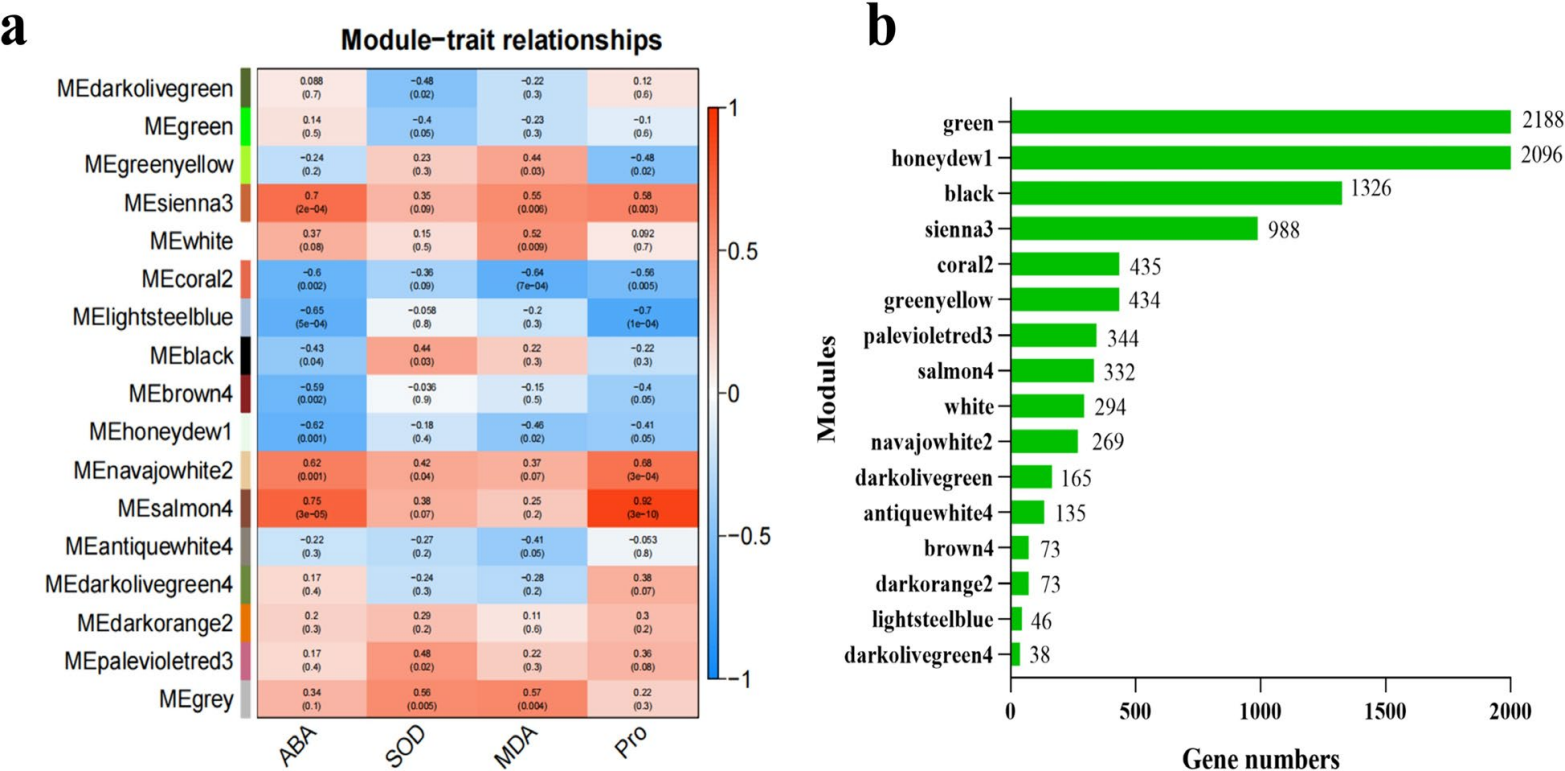
Weighted gene coexpressionnetwork analysis (WGCNA) of 12,892 DEGs obtained from all pairwise comparisons. **a** Module–trait correlations and corresponding p-values. The numbers in each cell represent the correlation coefficients and correlation significance levels (in parentheses). The color of the cell reflects the degree of correlation. These traits correspond to the four physiological indexes mentioned above. **b** The number of genes for each module

To reveal the differences in the gene regulation of the drought response in sunflower varieties with contrasting drought tolerances, weighted gene coexpression network analysis (WGCNA) was performed on the 12,892 DEGs (Fig. 8). Firstly, all the samples were clustered and it was found that the clustering was good, and no outliers were found (Supplementary Fig. 4). The soft threshold power of 14 (β = 14) was selected according to the preconditions of approximate scale-free topology (Supplementary Fig. 5a). Additionally, a module–trait relationship analysis was performed using module eigengene and physiological data for the two genotypes at 0 and 72 h. We set a module similarity threshold of 0.25 and a minimum number of genes within a module of 30 to classify the modules, and 17 merged coexpressed gene modules were identified (Fig. 8a). As shown in Fig. 8, the Sienna 3 module (containing 988 genes) was positively associated with ABA content, with a correlation coefficient (r) of 0.7 (*p* = 2 × 10^−4)^. The Navajo white 2 modules (containing 269 genes) were positively associated with ABA and Pro contents, with correlation coefficients (r) of 0.62 (*p* = 0.001) and 0.68 (*p* = 3 × 10^−4^), respectively. Salmon 4 modules (containing 332 genes) were positively associated with ABA and Pro contents, with correlation coefficients of 0.75 (*p* = 3 × 10^−5^) and 0.92 (*p* = 3 × 10^−10^), respectively. The coral 2 module (containing 435 genes) was negatively associated with ABA and MDA contents, with correlation coefficients of -0.6 (*p* = 0.002) and -0.64 (*p* = 7 × 10^−4^), respectively. Lightsteelblue modules (containing 46 genes) were negatively associated with ABA and Pro contents, with correlation coefficients of -0.65 (*p* = 5 × 10^−4^) and -0.7 (*p* = 1 × 10^−4^), respectively (Fig. 8a, b). This phenomenon may indicate possible correlations between genes that determine different drought resistance traits. Moreover, these results revealed the physiological and transcriptional differences between the leaves and roots of K55 and K58 in response to drought stress.

### GO enrichment analysis and KEGG pathway analysis of genes in each module

To reveal the functional differences of DEGs in the five significant modules, we performed GO-term enrichment analysis (Supplementary Fig. 6). The genes of each module were divided into three categories: biological process (BP), molecular function (MF), and cellular component (CC). We selected the most significant (*p* < 0.01) GO term for the analysis (Supplementary Fig. 6). After redundancy, genes in the Sienna 3 module were enriched in 63 GO terms, of which 29 were in BP, 29 were in MF, and 5 were in CC. The top three significant GO terms ranked by p value for BP were GO: 0006468 (protein phosphorylation), GO: 0000302 (response to reactive oxygen species), and GO: 0048544 (recognition of pollen). Genes in the navajowhite 2 module were enriched in 36 nonredundant GO terms, including 15 in BP, 3 in CC, and 29 in MF. The top three significant GO terms for BP were GO: 0006012 (galactose metabolic process), GO: 0009631 (cold acclimation), and GO: 0009737 (response to abscisic acid). After redundancy, genes in the salmon 4 module were enriched in 53 GO terms, of which 33 were in BP, 17 were in MF, and 3 were in CC. The top three significant BP-related GO terms were 0060918 (auxin transport), 0009251 (glucan catabolic process), and 0099402 (plant organ development). The genes in the coral 2 module were enriched in 58 nonredundant GO terms, including 25 in BP, 8 in CC, and 25 in MF. The top three significant GO terms for BP were GO: 0006334 (nucleosome assembly), GO: 0006325 (chromatin organization), and GO: 0010088 (phloem development). The genes in the lightsteelblue module were enriched in 18 nonredundant GO terms, including 7 in the BP category, 4 in the CC category, and 7 in the MF category. The top three significant GO terms for BP were GO: 0006412 (translation), GO: 0000027 (ribosomal large subunit assembly), and GO: 0000494 (box C/D snoRNA 3'-end processing).

To understand the function of genes in different modules more comprehensively, we also conducted a KEGG pathway enrichment analysis (Supplementary Table 3). Pathway analysis revealed that four of the five modules were enriched in a total of 19 pathways, while the lightsteelblue module was not enriched. Among them, the plant hormone signal transduction pathway was shared by the sienna 3 and navajowhite 2 modules. In addition, starch and sucrose metabolism pathways were specifically and significantly enriched in the salmon 4 and coral 2 modules. These findings indicate that these two pathways play a central roles in the sunflower response and resistance to drought stress.

**Fig. 9:**
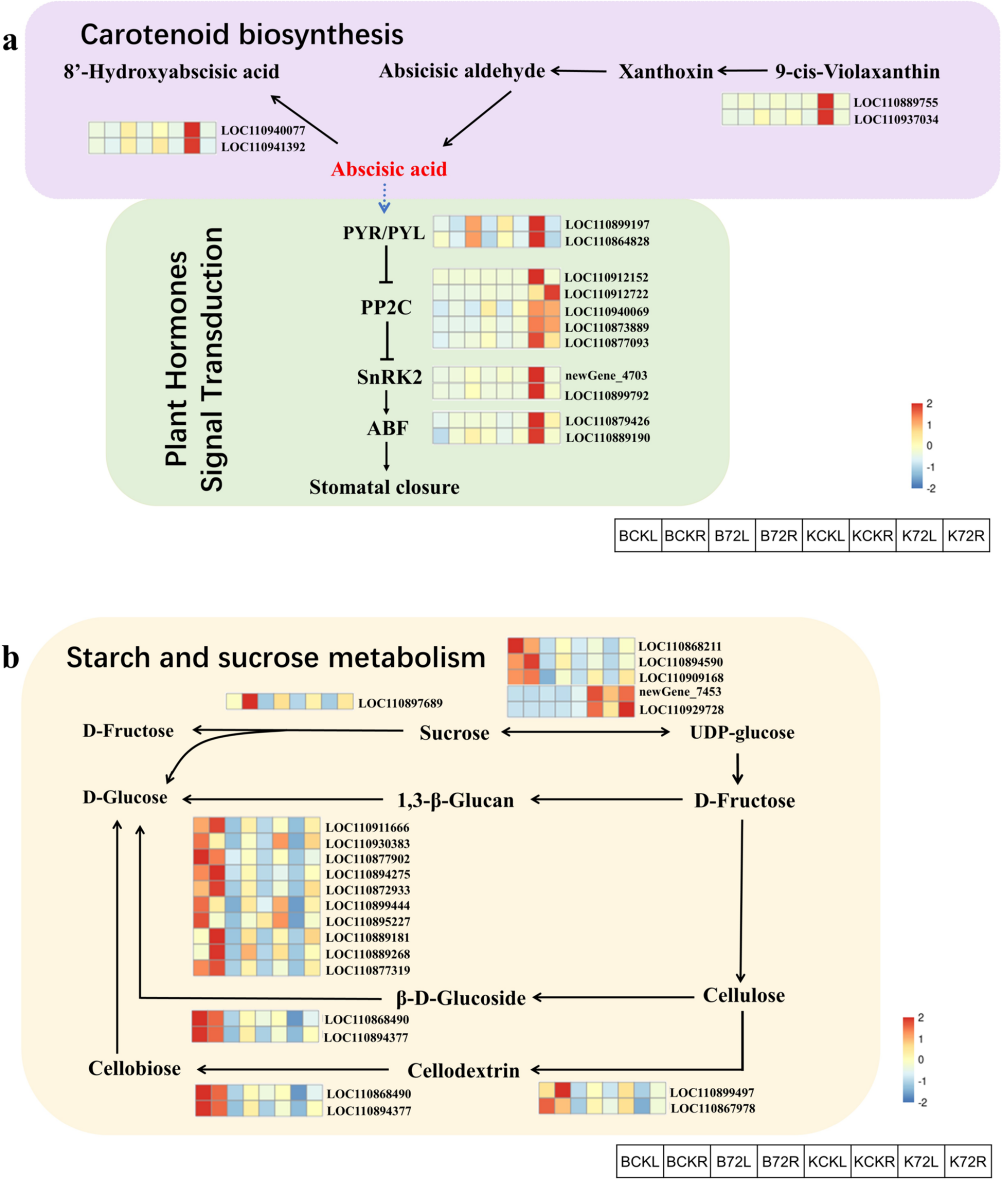
Metabolic network diagram in response to drought stress in sunflower. **a** ABA synthesis and signalling pathways. **b** Starch sucrose metabolic pathway

To further elucidate the mechanism underlying the response of sunflower to drought stress, we analysed the expression of genes related to the two metabolic pathways mentioned above. A total of 15 genes were enriched in the ABA synthesis and signal transduction pathways in both the sienna 3 and navajo_white 2 modules. As shown in Fig. 9a, all these genes were upregulated in the leaves after 72 h of drought stress in K58. However, the expression of these genes did not significantly change in K55. The above results indicate that the strongly drought-tolerant germplasm K58 can synthesize ABA faster in response to drought stress after exposure to drought stress. While salmon 4 and coral 2 had a total of 20 genes enriched in starch and sucrose metabolic pathways, we found that most of the genes were downregulated after 72 h of drought stress, except for the genes *Helianthus_annuus_newGene-7453* and *LOC110929728* (Fig. 9b). By gene function annotation, we found severalsucrose synthases, indicating that drought stress has an effect on sucrose synthesis.

**Fig. 10:**
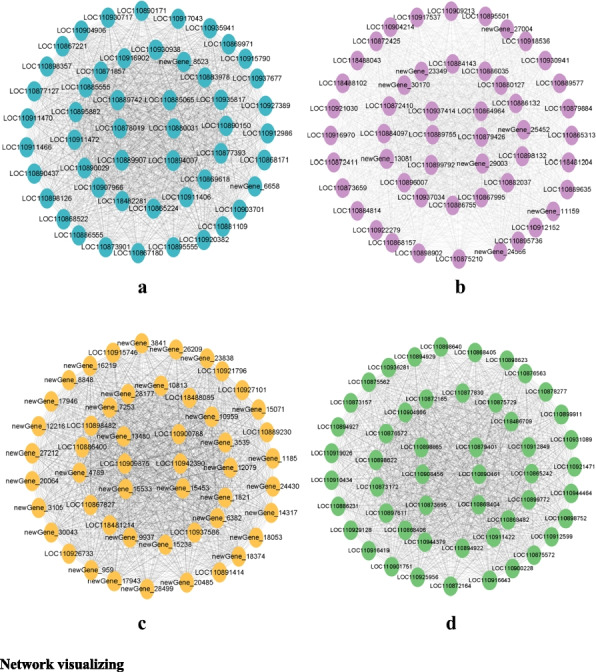
The top 50 genes in four main modules are calculated by MCC algorithm of cytohubba. **a** Top 50 genes in sienna 3module. **b** Top 50 genes in navajowhit 2 module. **c** Top 50 genes in salmon 4 module. **d** Top 50 genes in coral 2 module

The cytoHubba plugin identified the top 50 central genes in the 4 modules (Fig. 10). Genes in the sienna3 module were mainly annotated for different functions, most of which were protein kinases. The genes in the salmon4 module were mainly novel genes and enzymes of unknown function, such as isoforms and reductases. Most of the genes in the navajowhite2 module are related to transcription factors and protein phosphatases, such as *LOC110879426* (*ABI5*, belonging to the bZIP family) and *LOC110899792* (*SAPK3*). The coral 2 module is mainly related to histones.

### Verification of RNA-seq data by RT‒qPCR

**Fig. 11:**
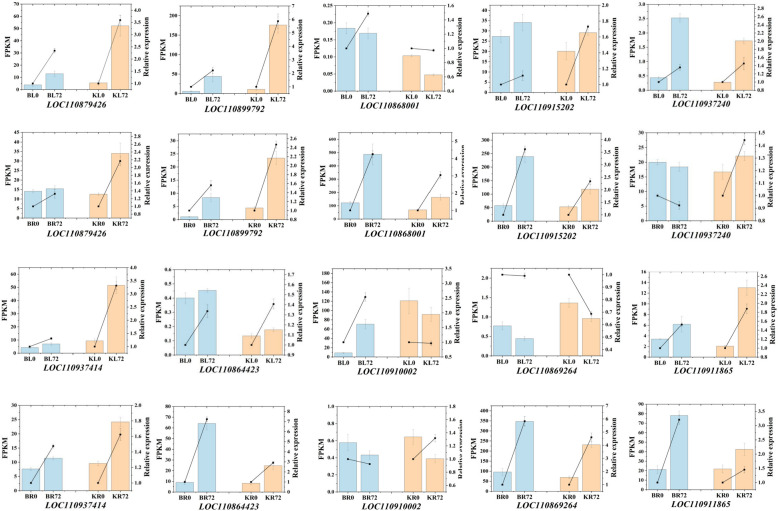
RT-qPCR analysis of 10 drought stress-associated DEGs in sunflower leaf and root samples at different stress time points. Bars with standard error indicate the average FPKM values of the samples at different stress time points. Scatter plots on the bars indicate the corresponding relative expression levels determined by RT-qPCR from three independent biological replicates using the 2^−∆∆Ct^ method

To verify the accuracy and reliability of the RNA-seq data, we performed quantitative real-time PCR (RT-qPCR) on 10 randomly selected genes (Fig. 11). As shown in the figure, the RNA-seq and RT-qPCR data showed high (90%) agreement in terms of the relative expression levels of the genes. These results indicate that the RNA-seq data are credible and accurate and can be used to identify candidate genes for further functional studies.

## Discussion

Sunflower plants are drought-resistant and nutrient deficient-tolerant because of their well-developed root system. However, since it is often grown in arid and semiarid regions of northern China [40], sunflower is often subject to water limitations during growth [41]. Therefore, breeding drought-resistant sunflower varieties is an effective way to overcome water limitations under drought conditions. In addition,screening sunflower drought- resistancerelated genes and isolating and characterizing them are the keys to revealing the molecular mechanism of drought resistance and improving the drought resistance of sunflower [42]. Previous studies have shown that the effect of drought on sunflower varies according to variety and tissue specificity. However, many studies have been conducted using only one genotypic material or a certain tissue of two genotypes. Studies combining the two are lacking, so elucidating the mechanisms of response to drought stress among different genotypes and different tissues of sunflower is lacking. We analysed the physiological changes in the seedling stage of sunflower plants under drought stress and the transcriptional profilesof sunflower seedlings under drought stress.

Overall, the number of DEGs detected in K55 was greater than that in K58, indicating that drought-sensitive genotypes are more vulnerable or highly sensitive to drought stress at the molecular level than drought-tolerant genotypes. These findings are similar to those of drought stress response studies in other plants [43–45]. The reason may be that drought-sensitive genotypes undergo greater changes in physiological and biochemical traits and transcript levels when mitigating the effects of stress conditions compared to those of drought-tolerant lines [46].

### Physiological and biochemical responses to drought stress in sunflower

Drought is currently one of the major abiotic stresses that severely constrains crop growth and development and yield components, thereby affecting agricultural production [47]. To survive and grow under drought stress, plants have evolved a complex response mechanism. Abscisic acid (ABA), as a typical stress hormone, mediates the general adaptive response of plants to drought and is the “first messenger” that initiates the expression of resistance genes in plants. The content of ABA in plants increases significantly during drought stress [48]. Our study revealed that the ABA content in the leaves and roots of both materials increased after drought stress, but the time to reach the maximum value differed between the materials. Overall, the ABA content of K58 was consistently greater than that of K55, suggesting that drought-tolerant germplasm can accumulate ABA faster in a short period of time to withstand drought stress. In addition, the leaves of both materials contained higher levels of ABA than did the roots when compared to the leaves and roots. This may be because ABA is usually produced in roots and transferred to leaves especially under drought conditions [49, 50]. MDA is the main product of lipid peroxidation in plant cell membranes under adverse stress conditions, and its level is related to the integrity of cell membranes. It can change the structure of membrane lipid molecules, thus inhibiting protein synthesis [51]. Under drought stress, the MDA content in the leaves and roots of both materials increased to different degrees. The MDA content in K58 was greater than that in K58 under the same drought stress time point except for leaf stress at 0 h. This result indicated that K55 suffered more oxidative damage and was more sensitive to drought stress than K58. The antioxidant enzyme protection system is an effective way for plants to defend against reactive oxygen damage, in which the SOD protective enzyme has a key regulatory role in eliminating H_2_O_2_. In our study, the SOD activity in the leaves and roots of K58 peaked at 48 and 24 h of stress, respectively. However, the activity decreased compared with that of K55, which indicated that the drought-tolerant material could rapidly accumulate antioxidant enzymes to scavenge ROS after drought stress. It is well known that proline acts as a signalling compound in the drought stress response by accumulating osmoregulation to maintain cell expansion in response to drought stress. In this study, the proline content in the leaves and roots of both K55 and K58 increased to different degrees with increasing drought duration. The proline content in K58 was greater than that in K55 during the same period. Curiously, from 0 to 48 h, the proline content of both materials did not change much, while at 72 h, there was a significant increase in the proline content in K58. This indicates that osmotic adjustment occurred later in the drought-tolerant material than in the less drought-tolerant material, which is consistent with the findings of a previous study [52]. This is because drought-tolerant materials have a greater ability to absorb water in the root system when subjected to drought stress. Therefore, plantscan maintain a elatively high water potential and thereby experience osmotic stress later.

### Differences in the number of DEGs and enriched GO terms between the two genotypes and tissues

When comparing the response of the two tissues to drought, we found that drought affected the gene expression of more genes in leaves than in roots, a result that was consistent in both genotypes and a similar pattern was previously observed in sunflower [25], where the number of DEGs was approximately four times greater in leaves than in roots. Leaf growth is more sensitive to drought stress than root growth [53]. In addition, our study revealed that drought treatment downregulated the expression of more genes. Under drought stress, the drought-sensitive material K55 had more DEGs, which is similar to the findings of Janiak et al. on the root transcriptome [54]. GO analysis of DEGs revealed that the DEGs identified after mild drought belonged to similar biological and functional categories as the DEGs identified after severe drought stress [55]. However, more DEGs were found in each GO category when the stress was stronger, and more DEGs were found in drought-sensitive genotypes. This is because the transcriptome of drought-sensitive genotypes was more profoundly altered than that of drought-tolerant genotypes, especially after severe stress. In contrast, Wu Yang et al. [56] reported the opposite conclusion, which may be the difference caused by different stress intensities and stress durations.

### Drought leads to reduced leaf photosynthesis

In green plants, photosynthesis in leaves is one of the first physiological processes affected by drought conditions [57]. When plants recognize changes in soil water deficit conditions under drought stress and transmit water deficit signals from the root system to the leaves through ABA accumulation [58], ABA promotes stomatal closure and inhibits stomatal opening, thereby reducing transpiration and water loss [59], with a concomitant reduction in gas exchange. In our study, the downregulated DEGs in K58 leaves were significantly enriched in photosynthesis-related pathways. This suggests that photosynthesis is inhibited under drought stress. Importantly, the ABA content in K58 leaves was significantly greater than that in K55 leaves after 72 h of drought stress. This suggests that, compared with K55, K58 was able to accumulate ABA in the fastest possible time after being subjected to drought stress, thereby promoting stomatal closure and reducing plant water loss. However, stomatal opening was not measured in this study. Normally, photosynthesis is weakened under drought conditions, which leads to an increase in oxygen in the plant, and, subsequently, photorespiration is enhanced to consume more than oxygen and reduce cellular damage. However, MapMan analysis in our study showed that some enzymes in the photorespiratory pathway in K58 were downregulatedafter drought stress, impairing photorespiration. It has been suggested that a reduction in photosynthesis has an impact on photorespiration [60], so this needs to be further investigated.

### Changes in carbohydrate metabolism in sunflower under drought stress

Carbohydrates are the main products of photosynthesis, and carbohydrate metabolism regulates sugar synthesis and conversion as well as carbon partitioning. Carbohydrate metabolism has been reported to play an important role in the response to drought stress, and starch and sucrose metabolism are associated with expansion maintenance [12, 61]. However, drought stress tends to disrupt carbohydrate metabolism in plants [62]. In our study, downregulated genes were enriched in the “biology” category of carbohydrate metabolism in both leaves and roots of both materials, but the number of downregulated genes was greater in leaves than in roots. The number of downregulated genes was greater in leaves than in roots, probably because the downregulation of photosynthesis in leaves leads to changes in carbohydrate metabolism [63]. However, the response of the root system to water deficit depends mainly on changes in the osmotic potential of carbohydrate metabolism, since carbohydrates in the root system are largely supplied by photosynthesis in the stem [64].

In this study, we found that both the salmon 4 and coral 2 module KEGG pathways were enriched in the starch sucrose metabolism pathway. Many previous studies have shown that starch and sucrose metabolic processes play important roles in the plant response to drought stress [65–68]. Glucose and sucrose are important soluble sugars for maintaining cellular osmotic potential [69, 70]. Sucrose synthase (SuSy) is a glycosyltransferase that catalyses the production of sucrose from UDP-glucose and plays a key role in sugar metabolism [71]. Leaf sucrose is converted to reducing sugars under drought stress by sucrose synthase and convertase [72]. Some sucrose synthase genes in our study were significantly downregulated in weakly drought-tolerant K55 leaves, whereas they were not significantly changed in K58 leaves. This is consistent with the findings obtained by Zhou et al. in soybean roots [73]. Another study showed that drought stress increased the transcript level of *GmSusy45* in soybean leaves [74]. The expression levels of sucrose synthase were different at different filling stages, indicating that there were temporal and spatial differences in the expression of this gene. In addition, inactive ABA in Arabidopsis leaves is hydrolysed by β-glucosidase, which then releases large amounts of ABA to improve drought tolerance [75]. In this study, the β-glucosidase gene was more downregulated in K55 after drought stress, which may also be one of the reasons why its ABA content was lower than that of K58.

### Ribosome pathway

Translational regulation is a key stage in the regulation of gene expression [76], and the ribosome, as the centre of translation, is the main site for the synthesis of many regulatory substances [77]. Many studies have shown that translational regulation plays an important role in the plant response to drought stress. Some studies have shown that drought stress increases ribosome expression, which is contrary to our findings. Our study revealed that downregulated DEGs in leaves were enriched in the BP class in the translational pathway and in the cytoplasmic ribosomal small subunit, cytoplasmic ribosomal large subunit, and ribosomes of the CC class. KEGG was also enriched for ribosomes. The genes enriched in this pathway mainly encoded 40S, 50S, and 60S ribosomal subunits, suggesting that drought has an effect on ribosome assembly. This is in agreement with the findings obtained in studies on *Auricularia fibrillifera* [78]. Tripathi et al. suggested that the translation process is energy-consuming, and the downregulation of translation under drought stress saves energy [79]. However, the response mechanism of genes in the ribosomal pathway under drought stress is still unclear, and further investigation is needed to elucidate the underlying mechanism involved.

### Changes in ABA biosynthesis and metabolism in sunflower under drought stress

It is well known that endogenous ABA levels are elevated in plants under drought conditions. In our study, ABA levels were significantly greater in the strongly drought-tolerant sunflower germplasm K58 than in the weakly drought-tolerant germplasm K55 after drought stress exposure. It is well known that ABA levels in plants are regulated by ABA biosynthesis and degradation pathways [80]. In plants, the carotenoid pathway is the main pathway for ABA synthesis [81]. The upregulated genes expressed in the roots of K58, a strongly drought-tolerant germplasm, were enriched in the carotenoid biosynthesis pathway in this study. In addition, this pathway was also enriched in the navajowhite2 module, which is significantly related to ABA. This suggests that when sunflower is subjected to drought stress, roots of strongly drought-tolerant germplasms can synthesize ABA faster. Some ABA synthesis and degradation-related genes in this pathway were upregulated and expressed in K58, among which *LOC110912807* encodes β-carotene hydroxylase 2, which is homologous to Arabidopsis *AT4G25700*. β-carotene hydroxylase (BCH) is a key enzyme in the plant carotenoid biosynthetic pathway that catalyses the synthesis of zeaxanthin from β-carotene in plants via the intermediate product β-cryptoxanthin [82, 83]. Rice encodes *BCH1*, which promotes resistance to drought and oxidative stress by increasing rice lutein and ABA levels [83, 84]. *LOC110895635* encodes a zeaxanthin epoxidase that is homologous to Arabidopsis *AT5G67030*. Zeaxanthin epoxidase (ZEP) catalyses the conversion of zeaxanthin to zeaxanthin, a key reaction in the biosynthesis of ABA, which is important for adaptation to environmental stresses, especially drought [85]. Overexpression of *AtZEP* enhanced plant tolerance to drought stress [86]. In ABA biosynthesis, β-carotene is first converted to zeaxanthin by the enzyme *CHY2*, and then the epoxidation of zeaxanthin and anthracenoxanthin to zeaxanthin is catalysed by zeaxanthin epoxidase (ZEP/ABA1) [87]. Then, cisneoxanthin can be oxidatively cleaved by 9-cis-epoxycarotenoid dioxygenase (NCED), thereby synthesizing ABA [81]. In our study, *LOC110889755* and *LOC110937034* encoded the 9-cis-epoxycarotenoid dioxygenases NCED1 and NCED2, respectively. By comparison, these two genes were found to be homologous to *Arabidopsis thaliana AT3G14440* and *AT1G30100* (Supplementaary Table 4). Previous studies have shown that members of this subfamily of proteins are rate-limiting enzymes in the biosynthesis of ABA hormones [88] and respond positively to drought stress. Overexpression of the brassinosteroid *PvNCED1* in wild tobacco increased the ABA content and improved the tolerance of tobacco plants to drought stress [89]. The same conclusion has been reached in studies on wheat and watermelon [90, 91].

The *CYP707A* gene encodes ABA 8′-hydroxylase, a key enzyme in the catabolism of ABA [92, 93]. Under drought conditions, endogenous ABA levels are elevated in plants, and the enzyme *CYP707A* controls endogenous ABA levels; overexpression of *CYP707A* leads to a decrease in ABA levels [94, 95]. In this study, the genes encoding abscisic acid 8'-hydroxylase *CYP707A1* and *CYP707A2* were upregulated after drought stress and were more upregulated in the weakly drought-tolerant germplasms. These findings suggest that these genes are involved in the response of sunflowers to drought stress, which is similar to the results of many studies. *CYP707A* genes were upregulated under drought stress in maize, and *CYP707A1* and *CYP707A2* genes were highly expressed in Arabidopsis under osmotic stress and drought stress [96]. Two key enzymes involved in ABA degradation were previously reported to exhibit specific expression patterns, namely, *CYP707A3* in ductal tissues and *CYP707A1* in stomata [97], suggesting that the regulation of this gene family may be tissue specific.

### Analysis of phytohormone signalling and protein kinase response to drought stress

Reversible protein phosphorylation, one of the most prevalent posttranslational modifications in eukaryotic organisms, plays a paramount role in plant defence against external stresses. Our study revealed that most of the upregulated DEGs in the leaves were enriched in protein phosphorylation and dephosphorylation in biological pathways. Protein kinases can phosphorylate specific substrates, which are key components of the plant drought stress response [98]. In our study, a large number of protein kinases, including cysteine -rich receptor-like protein kinases, G-type lectin S receptor-like serine/threonine protein kinases, and the osmotic stress/ABA-activated protein kinase 2 and 3 (*SAPK2*/*SAPK3*) family were found to be upregulated in leaves under drought stress. Changes in protein phosphorylation in plants are induced by ABA [99]. By visualizing the MapMan metabolic network, we found that phytohormones are predominantly enriched in the ABA pathway. The ABA signalling pathway plays a central role in the plant response to drought stress and salt stress [100–102]. ABA signalling genes involved in the water stress response include *PYR*/*PYL*/*RCAR*, *PP2C* (negative regulator), and *SnRK2* (positive regulator) [64]. Under drought stress, the complex formed by PYR/PYL/RCAR and PP2C inhibits the dephosphorylating activity of *PP2C*, which activates *SnRK2* and leads to the closure of stomatal pores [103]. The ABA receptor *PYL9* has been proven to promote drought resistance and leaf senescence [104]. In our study, we found that a number of genes encoding *PYL* and *PP2C* were upregulated in sunflowers under drought stress, and the functions of these genes have been frequently reported. For example, the ABA receptor *PYL9* was shown to promote drought tolerance and leaf senescence [105]. The activation of *PP2C* genes in grapes in response to drought stress suggests that *PP2C* plays a major role in stress tolerance, particularly in the regulation of stomatal responses in response to transpirational losses [106]. In addition, *SnRK2* is an important regulator of ABA signalling during drought stress [107]. In this study, *LOC110899792* and *Helianthus_annuus_newGene_4703* both belong to the *SnRK2* family and encode *SAPK3* and the protein kinase CAMK-OST1L, respectively. These genes are highly expressed in K58 leaves and are homologous to Arabidopsis *At1g60940* and *At5g08590* (Supplementaary Table 4), respectively. *SAPK3-1* and *SAPK3-2* exhibit enhanced drought tolerance under drought stress, including reduced survival, increased water loss, and increased stomatal conductance [108]. Moreover, the expression of *OsSAPK3* in rice is regulated by drought, NaCl, PEG, and ABA. *OsSAPK3* mutant seeds (*sapk3-1* and *sapk3-2*) showed reduced hypersensitivity to exogenous ABA, suggesting that the gene is responsive to both endogenous and exogenous ABA. Furthermore, in wild soybean, the expression of the GSSR K (G-type lectin S receptor-like serine/threonine protein kinase) gene is induced by salt, drought, and ABA [109]. Cysteine-rich receptor-like protein kinases have also been suggested to control seedling growth arrest and stomatal closure in response to drought [110] and can sense drought by upregulating the G-type lectin receptor-like serine/threonine protein kinase SRK [109]. Taken together, these findings suggest a role or protein kinases in sunflowers in response to drought through ABA signalling.

### Major transcription factors associated with drought tolerance in sunflower seedlings

Transcription factors are key players in plant regulatory networks in response to unfavourable environmental stresses. In the present study, many transcription factors were predicted by trans-iTAK, among which the *AP2/ERF-ERF* family had the most members, followed by *MYB*, *bHLH*, *C2H2*, and *WRKY*. Many studies have indicated that these transcription factors play important roles in the plant response to drought stress [111]. One study revealed that most *AP2/ERF* transcription factors exhibit root-specific expression [112], which is similar to the results of our study, in which we detected a greater number of *ERF* transcription factors in roots than in leaves. Some recent studies have shown that some *MYB* genes in sunflowers are highly expressed under drought stress [113]. According to our results, *MYB* genes were more downregulated in the leaves than in the roots. In addition, our study revealed that the expression of *MYB* genes was tissue specific. Similarly, Li et al. reported that the transcripts of several *MYB* genes were initially highly expressed in roots and then decreased with increasing stress concentration [114]. Therefore, this result may be the result of different stress intensities. Li et al. reported that the *HabHLH159* and *HabHLH024* genes were highly expressed under drought stress [115]. Our study results are similar to the findings of Wu et al. [26]. A large number of *bHLH* family genes are downregulatedin leaves under drought stress. However, a study on foxtail cereals indicated that *bHLH* genes were biased for expression in root and fleshy tissues [116], which might be caused by the tissue specificity of this transcription factor family in different plants. It has been reported that increased overexpression of both *OsbHLH148* and *SlbHLH96* improved drought tolerance in plants, whereas silencing of *SlbHLH96* in tomato reduced drought tolerance, which was related to ROS metabolism. Further studies revealed that *SlbHLH96* binds directly to the cis-element in the *SlCYP707A2* promoter and downregulates its transcription. This leads to an increase in endogenous ABA levels, which in turn regulates the expression of genes associated with the ABA response [117]. Importantly, most grape *bHLH* gene promoters were found to contain *MYB* binding sites involved in the drought response in grape [118], suggesting that there is some coordination between these transcription factors, which deserves further in-depth study. In addition, members of the bZIP family are important transcription factors involved in the ABA signalling pathway, and ABI5 is an important member of the bZIP family [119]. ABI5 expression is affected by drought and salt stress [120], and in *Arabidopsis thaliana*, ABI5 and ABFS/AREB are key ABA-dependent signalling factors involved in abiotic stress tolerance [121]. In this study, we found that the expression of *LOC11087942*6, a gene encoding the ABI5 protein, in K58 leaves increased 3.24-fold after drought stress and was enriched in the ABA signalling pathway. This finding was confirmed by RT‒qPCR analysis. This gene may beinvolved in the response of sunflower seedlings to drought stress. This gene is homologous to *Arabidopsis thaliana AT1G45249* (Supplementary Table 4). In Arabidopsis, this gene encodes abscisic acid response element binding Factor 2, and several studies have reported that this gene is responsive to salt stress [122] and drought tolerance [123]. These results reveal the important role of transcription factors in the response to ABA and drought stress in sunflowers.

## Conclusion

In this study, the physiology and transcriptome of the leaves and roots of two sunflower materials under drought stress were analysed. Analysis of variance (ANOVA) for physiological traits showed that the greatest differences were found at 0 h and 72 h. Transcriptome data showed that the sensitive lines experienced greater changes in transcript levels before and after drought stress than did the drought-tolerant lines. GO and KEGG enrichment analyses combined with WGCNA revealed “photosynthesis”, “ribosome and translation”, “starch-sucrose metabolic pathway” and “phytohormone signalling and protein phosphorylation” in response to drought stress. In addition, many TF genes are involved in the regulatory network under drought stress. These findings not only contribute to our understanding of the potential molecular mechanisms of drought tolerance in sunflower plants. Our further study revealed the role of hub genes in the drought tolerance regulatory network, laying the foundation for breeding drought-tolerant varieties. Unfortunately, we did not measure the photosynthesis-related parameters of the sunflower leaves before and after drought in this study, which prevented us from making our study more comprehensive. In addition, it is very important that we do not perform further functional validation of the screened candidate genes, which will be the focus of our next study.

### Supplementary Information

**Supplementary Material 1.**

**Supplementary Material 2.**

## Data Availability

Sequence data that support the findings of this study have been deposited in the European Nucleotide Archive with the primary accession code PRJNA1041959.

## Abbreviations

ANOVA: Analysis of variance
ELISA: Enzyme-linked immunoassay adsorption
SOD: Superoxide dismutas
ABA: Abscisic acid
DEGs: Differentially expressed genes
FPKM: Fragments per kilobase of transcript per million mapped reads
GO: Gene Ontology
KEGG: Kyoto encyclopedia of genes and genomes
PCA: Principal component analysis
RNA-seq: RNA sequencing
ROS: Reactive oxygen species
RT-qPCR: Quantitative real-time PCR
WGCNA: Weighted gene coexpressionnetwork analysis
