# Supplementary material for: Comparative transcriptome and coexpression network analysis reveals key pathways and hub candidate genes associated with sunflower (Helianthus annuus L.) drought tolerance
Source: BMC Plant Biol. 2024 Mar 27;24:224. doi: 10.1186/s12870-024-04932-w (PMC10976745; doi:10.1186/s12870-024-04932-w)
Supplement: Supplementary file 1 — Supplementary Material 1. [file 12870_2024_4932_MOESM1_ESM.zip › Supplementary table/Supplementary table legends.docx]

**Supplementary table legends**

**Supplementary table 1.** RT-qPCR primer sequences.

**Supplementary table 2.** Summary of the sequence data from RNA sequencing.

**Supplementary table3** Significant Kyoto Encyclopedia of Genes and Genomes (KEGG) pathways of four significant WGCNA modules.

**Supplementary table 4.** List of Candidate Genes for Response to Drought Stress in Sunflower with Homology Comparison to Arabidopsis thaliana.
