# Supplementary material for: Comparative transcriptome and coexpression network analysis reveals key pathways and hub candidate genes associated with sunflower (Helianthus annuus L.) drought tolerance
Source: BMC Plant Biol. 2024 Mar 27;24:224. doi: 10.1186/s12870-024-04932-w (PMC10976745; doi:10.1186/s12870-024-04932-w)
Supplement: Supplementary file 1 — Supplementary Material 1. [file 12870_2024_4932_MOESM1_ESM.zip › Supplementary table/Supplementary table1.docx]

**Supplementary table 1 RT-qPCR primer sequences**

| Gene | Forward primer sequences | Reverse primer sequences | Product length（bp） |
| --- | --- | --- | --- |
| Ha18S | GATCGGAGTAATGATTAACAG | TTATGGTTGAGACTAGGACG | 175 |
| LOC110879426 | CGTCTGTATCGCCTGTGCCTTATG | TAATCATCCGCCTCTGCCTCCTC | 102 |
| LOC110899792 | GCTGGCAGATTCAGTGAAGACGAG | GGGCAATGTACGCTGGTGTTCC | 229 |
| LOC110937414 | GACCCACACGACCCTCAACTTG | CTCCCATTAACATGCCCACCTTCC | 75 |
| LOC110937240 | TCATCTCAACGCCGCATCATCAC | TCAGGCTCAGGCTTAGGCTCAG | 141 |
| LOC110864423 | TGGTGGTTCAAGTAACAGCCGATG | GACGAGTAGGTTGCGAGTGGTTATG | 222 |
| LOC110868001 | GCTTCACCATCACCACCACCTTC | GTCACTGTCACCACCCAAATCCG | 159 |
| LOC110911865 | TAGCAGCTTCACTCCTCTCGTCTC | TGGTTCCATGAGTTGGGCAGATTG | 230 |
| LOC110915202 | GCTCGGCTTCACTCGGTTCATC | GGCTTCGTCACAAGGGTTAGGC | 89 |
| LOC110910002 | GACCGACGATGAGCCACCAATG | CGAGGAGGAGAAAGGACGAACTTG | 181 |
| LOC110869264 | AAGTGGGTGTGTGAGGTCAGAGAG | CGCCATGTCAGCAGTCGGATATG | 78 |
