# Supplementary material for: Comparative transcriptome and coexpression network analysis reveals key pathways and hub candidate genes associated with sunflower (Helianthus annuus L.) drought tolerance
Source: BMC Plant Biol. 2024 Mar 27;24:224. doi: 10.1186/s12870-024-04932-w (PMC10976745; doi:10.1186/s12870-024-04932-w)
Supplement: Supplementary file 1 — Supplementary Material 1. [file 12870_2024_4932_MOESM1_ESM.zip › Supplementary table/Supplementary table2.docx]

| Samples | Clean reads | Clean bases | GC Content (%) | Q30(%) | Mapping Ratio (%) | Uniq Mapped Reads | Uniq Mapping Ration (%) |
| --- | --- | --- | --- | --- | --- | --- | --- |
| K0hL-1 | 19,730,047 | 5,905,251,482 | 44.93% | 94.01% | 91.35% | 34, 336, 384 | 87.02% |
| K0hL-2 | 24,959,336 | 7,470,500,320 | 45.04% | 93.47% | 91.27% | 43, 027, 616 | 86.20% |
| K0hL-3 | 22,425,361 | 6,712,653,770 | 45.38% | 94.27% | 91.76% | 38, 625, 417 | 86.12% |
| K72hL-1 | 24,608,809 | 7,364,241,790 | 44.33% | 94.30% | 89.84% | 42, 318, 667 | 85.98% |
| K72hL-2 | 19,625,132 | 5,873,106,042 | 44.09% | 93.93% | 90.34% | 34, 101, 858 | 86.88% |
| K72hL-3 | 20,211,199 | 6,048,339,074 | 44.26% | 93.99% | 90.32% | 35, 116, 404 | 86.87% |
| B0hL-1 | 22,022,576 | 6,590,218,428 | 45.33% | 94.47% | 91.30% | 38, 093, 440 | 86.49% |
| B0hL-2 | 23,448,209 | 7,015,743,714 | 45.68% | 94.26% | 91.55% | 40, 277, 713 | 85.89% |
| B0hL-3 | 20,778,668 | 6,217,534,880 | 45.52% | 93.95% | 91.41% | 35, 871, 752 | 86.32% |
| B72hL-1 | 20,134,057 | 6,025,277,204 | 44.76% | 92.81% | 88.97% | 34, 482, 159 | 85.63% |
| B72hL-2 | 21,709,897 | 6,496,883,472 | 44.71% | 93.86% | 90.47% | 37, 523, 943 | 86.42% |
| B72hL-3 | 23,023,452 | 6,889,800,084 | 44.53% | 94.35% | 90.46% | 40, 025, 653 | 86.92% |
| K0hR-1 | 21,109, 394 | 6,315,592,350 | 44.16% | 94.32% | 89.44% | 36, 123, 734 | 85.56% |
| K0hR-2 | 21,789,412 | 6,519,463,818 | 44.33% | 94.18% | 90.02% | 37, 360, 016 | 85.73% |
| K0hR-3 | 22,946,875 | 6,862,860,876 | 44.24% | 93.95% | 89.45% | 39, 289, 243 | 85.61% |
| K72hR-1 | 19,192,422 | 5,739,391,362 | 44.90% | 94.43% | 86.56% | 31, 715, 435 | 82.62% |
| K72hR-2 | 19,181,907 | 5,739,304,830 | 44.03% | 94.14% | 89.43% | 32, 697, 647 | 85.23% |
| K72hR-3 | 22,274,029 | 6,664,155,698 | 44.04% | 94.23% | 89.81% | 38, 300, 817 | 85.98% |
| B0hR-1 | 19,129,608 | 5,723,483,582 | 44.13% | 94.15% | 89.22% | 32, 870, 526 | 85.92% |
| B0hR-2 | 23,303,478 | 6,969,590,542 | 44.38% | 93.99% | 89.76% | 40, 215, 795 | 86.29% |
| B0hR-3 | 21,384,074 | 6,397,601,832 | 44.20% | 94.28% | 89.91% | 36, 918, 644 | 86.32% |
| B72hR-1 | 25,085,112 | 7,502,845,734 | 45.33% | 94.47% | 89.67% | 43, 221, 899 | 86.15% |
| B72hR-2 | 19,674,707 | 5,885,355,228 | 45.68% | 94.26% | 89.44% | 33, 830, 222 | 85.97% |
| B72hR-3 | 21,160,819 | 6,329,841,622 | 45.52% | 93.95% | 88.85% | 36, 227, 715 | 85.60% |

**Supplementary table 2 Summary of the sequence data from RNA sequencing**
