# Supplementary material for: Comparative transcriptome and coexpression network analysis reveals key pathways and hub candidate genes associated with sunflower (Helianthus annuus L.) drought tolerance
Source: BMC Plant Biol. 2024 Mar 27;24:224. doi: 10.1186/s12870-024-04932-w (PMC10976745; doi:10.1186/s12870-024-04932-w)
Supplement: Supplementary file 1 — Supplementary Material 1. [file 12870_2024_4932_MOESM1_ESM.zip › Supplementary table/Supplementary table3.docx]

| **Module** | **Pathway ID** | **Pathway** | **Number of**  **Genes** | **p-value** |
| --- | --- | --- | --- | --- |
| sienna 3 | ko04626 | Plant-pathogen interaction | 85 | 9.16×10-14 |
|  | ko00402 | Benzoxazinoid biosynthesis | 7 | 3.48×10-7 |
|  | ko00902 | Monoterpenoid biosynthesis | 6 | 1.89×10-3 |
|  | ko04075 | Plant hormone signal transduction | 36 | 4.60×10-3 |
|  | ko00603 | Glycosphingolipid biosynthesis-globo and isoglobo series | 3 | 7.51×10-3 |
|  | ko00760 | Nicotinate and nicotinamide  metabolism | 5 | 8.08×10-3 |
|  | ko03022 | Basal transcription factors | 6 | 8.54×10-3 |
| navajowhite | ko04075 | Plant hormone signal transduction | 14 | 2.00×10-3 |
|  | ko00280 | Valine, leucine and isoleucine degradation | 4 | 2.72×10-3 |
|  | ko04016 | MAPK signaling pathway-plant | 11 | 3.32×10-3 |
|  | ko00906 | Carotenoid biosynthesis | 3 | 3.93×10-3 |
|  | ko00052 | Galactose metabolism | 5 | 7.22×10-3 |
| salmon 4 | ko00500 | Starch and sucrose metabolism | 8 | 2.17×10-3 |
|  | ko00460 | Cyanoamino acid metabolism | 5 | 2.85×10-3 |
|  | ko00480 | Glutathione metabolism | 4 | 4.03×10-3 |
|  | ko00400 | Phenylalanine, tyrosine and  tryptophan biosynthesis | 3 | 6.18×10-3 |
| coral 2 | ko00500 | Starch and sucrose metabolism | 18 | 1.25×10-7 |
|  | ko00040 | Pentose and glucuronate  interconversions | 14 | 1.41×10-4 |
|  | ko00531 | Glycosaminoglycan degradation | 4 | 3.51×10-3 |

**Supplementary table 3** **Significant Kyoto Encyclopedia of Genes and Genomes (KEGG) pathways of four significant WGCNA modules**
