# Supplementary material for: Comparative transcriptome and coexpression network analysis reveals key pathways and hub candidate genes associated with sunflower (Helianthus annuus L.) drought tolerance
Source: BMC Plant Biol. 2024 Mar 27;24:224. doi: 10.1186/s12870-024-04932-w (PMC10976745; doi:10.1186/s12870-024-04932-w)
Supplement: Supplementary file 2 — Supplementary Material 2. [file 12870_2024_4932_MOESM2_ESM.zip › Supplementary figure/supplementary figure legends.docx]

**Supplementary figure legends**

**Additional file 1**: **Figure S1**. Volcano map of differentially expressed genes. Note: Red represents upregulated genes, green represents downregulated genes, and black represents no significant difference, with thresholds of (padj ≤ 0.01 and | log2(fold change) | ≥ 1) (a) DEG in the leaf blades of drought-stressed 72h K55 leaves. (b) DEG in the leaf blades of drought-stressed 72h K58 leaves. (c) DEG in the root system of drought-stressed 72h K55. (d) DEG in the root system of drought-stressed 72h K55.) DEG in drought-stressed 72h K58 root system.

**Figure S2**. GO enrichment plots for each differential group. (a) GO enrichment analysis of upregulated DEG in K55 drought-stressed 72h leaves. (b) GO enrichment analysis of upregulated DEG in K58 drought-stressed 72h leaves. (c) GO enrichment analysis of upregulated DEG in K55 drought-stressed 72h roots. (d) GO enrichment analysis of unregulated DEG in K58 drought-stressed 72h roots. (e) GO enrichment analysis of downregulated DEG in K55 drought-stressed 72h leaves. (f) GO enrichment analysis of downregulated DEG in K58 drought-stressed 72h leaves. (g) GO enrichment analysis of downregulated DEG in K55 drought-stressed 72h roots. (h) GO enrichment analysis of downregulated DEG in K58 drought-stressed 72h roots.

**Figure S3**. Plot of KEGG enrichment analysis for each differential groups. (a)KEGG analysis of uprelated DEG in leaves of K55 under drought stress for 72h. (b) KEGG analysis of up-regulated DEG in leaves of drought-stressed 72hK58. (c) KEGG analysis of up-regulated DEG in roots of K55 under drought stress 72h. (d) KEGG analysis of uprelated DEG in K58 drought-stressed 72h roots. (e) KEGG analysis of downregulated DEG in leaves of drought-stressed 72hK55. (f) KEGG analysis of downregulated DEG in leaves of drought-stressed 72hK58. (g) KEGG analysis of downregulated DEG in roots of drought-stressed 72hK55. (h) KEGG analysis of downregulated DEG in roots of drought-stressed 72hK58.

**Figure S4**. Clustering dendrogram of samples based on their Euclidean distance to detect outliers.

**Figure S5**. Soft-threshold selection of gene coexpression network and Hierarchical clustering tree. a. Soft-threshold selection. The left graph shows as the scale-free fit index (y-axis) for different soft thresholds (x-axis). The right graph shows the average degree of connectivity of the network under different soft thresholds (x-axis). b. Hierarchical clustering tree showing 11 coexpression modules identified by WGCNA. Different modules are marked with different colors. Each leaf of the cluster tree represents a gene.

**Figure S6**. GO enrichment analysis of five significant modules. All significant GO-terms presented in the heatmap have been de-redundant with the REVIGO online platform. (a)sienna3 module. (b)salmon4 module. (c) navajowhite2 module. (d)coral2 module. (e) lightsteelblue module
