# Supplementary figures and images for: Comparative transcriptome and coexpression network analysis reveals key pathways and hub candidate genes associated with sunflower (Helianthus annuus L.) drought tolerance
Source: BMC Plant Biol. 2024 Mar 27;24:224. doi: 10.1186/s12870-024-04932-w (PMC10976745; doi:10.1186/s12870-024-04932-w)

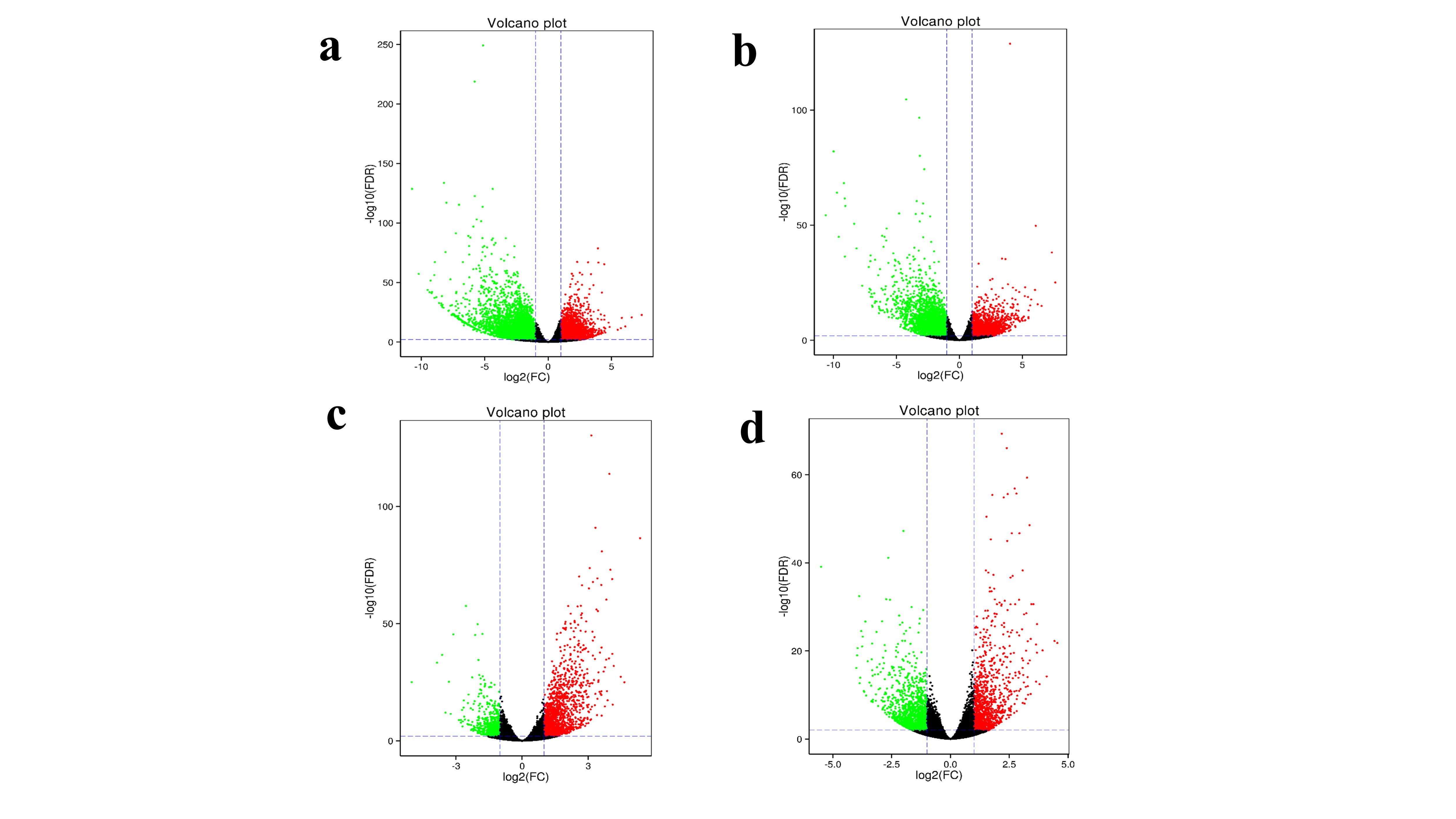

Supplement: Supplementary file 2 — Supplementary Material 2. [file 12870_2024_4932_MOESM2_ESM.zip › Supplementary figure/Supplementary figure 1.jpg]

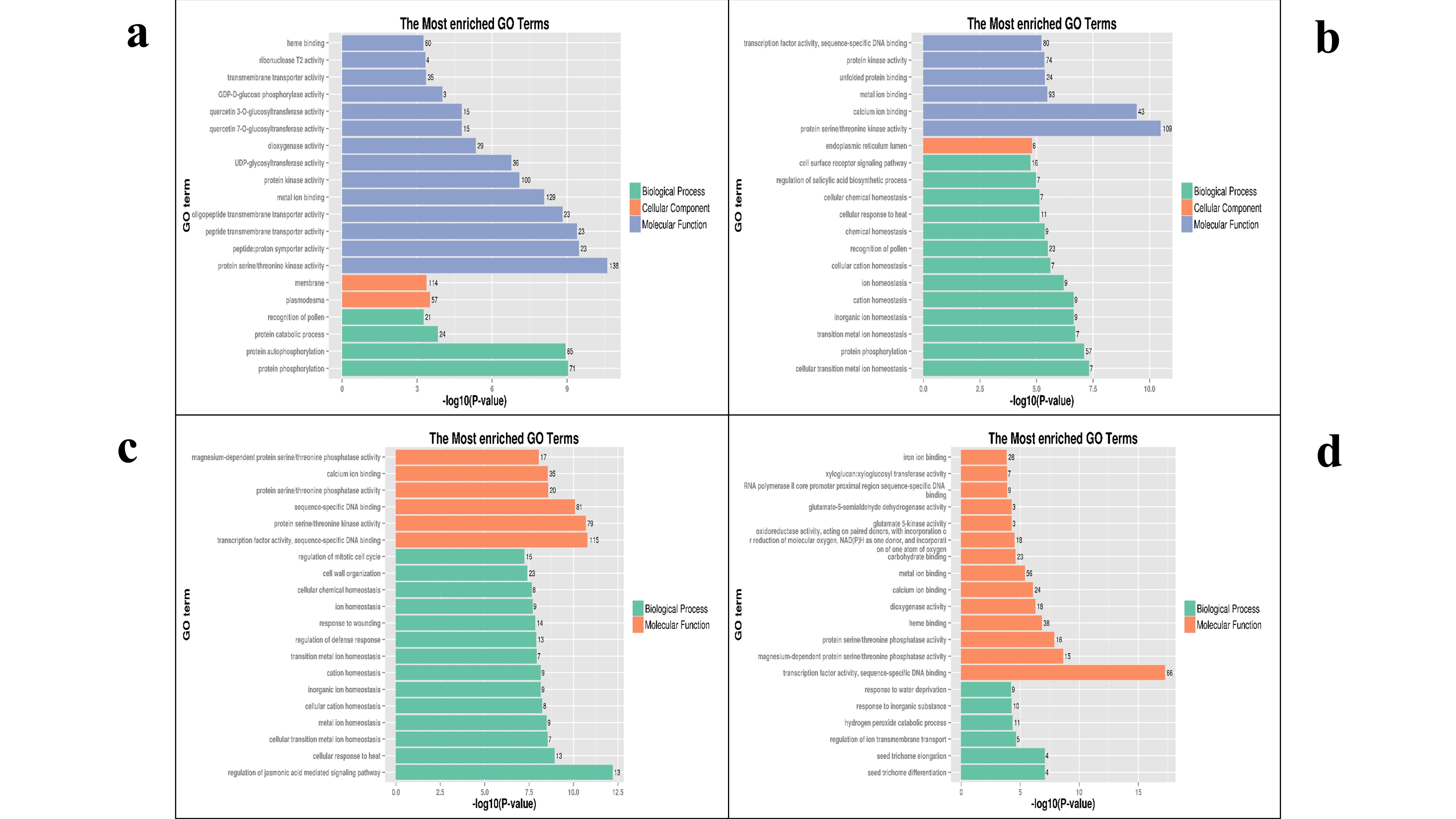

Supplement: Supplementary file 2 — Supplementary Material 2. [file 12870_2024_4932_MOESM2_ESM.zip › Supplementary figure/Supplementary figure 2 a,b,c,d.jpg]

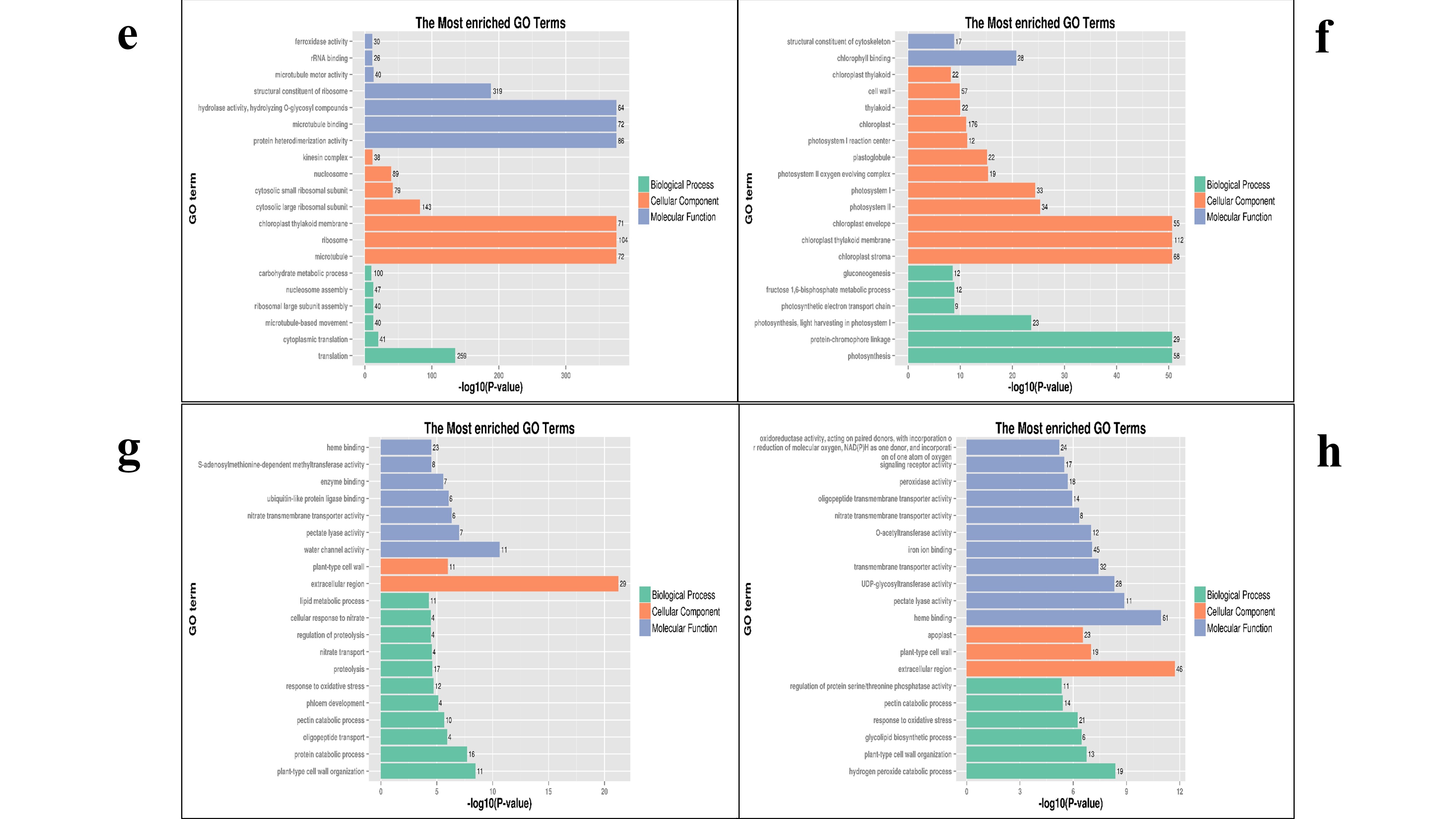

Supplement: Supplementary file 2 — Supplementary Material 2. [file 12870_2024_4932_MOESM2_ESM.zip › Supplementary figure/Supplementary figure 2,e,f,g,h.jpg]

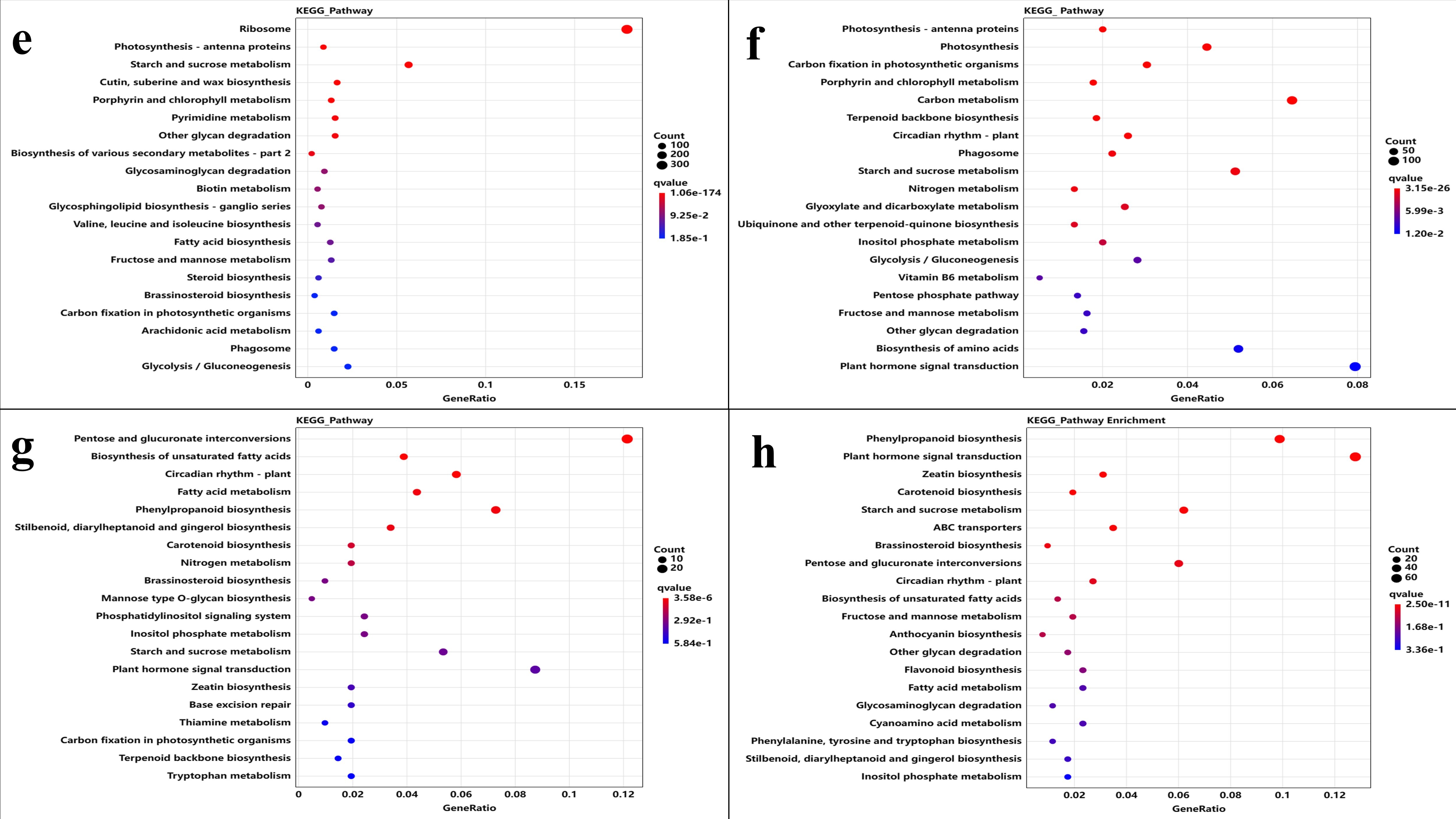

Supplement: Supplementary file 2 — Supplementary Material 2. [file 12870_2024_4932_MOESM2_ESM.zip › Supplementary figure/Supplementary figure 3,e,f,g,h.jpg]

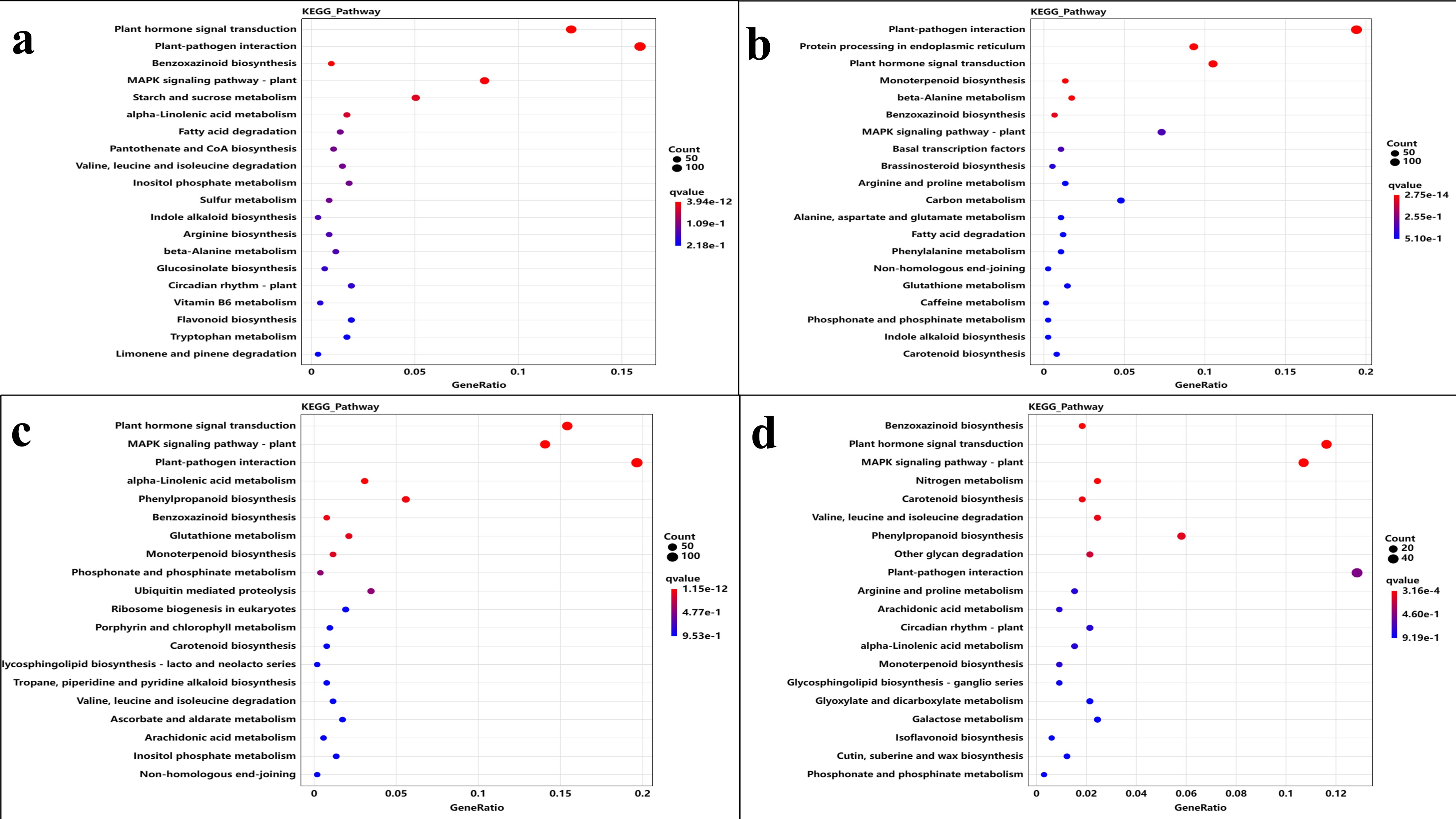

Supplement: Supplementary file 2 — Supplementary Material 2. [file 12870_2024_4932_MOESM2_ESM.zip › Supplementary figure/Supplementary figure 3a,b,c,d.jpg]

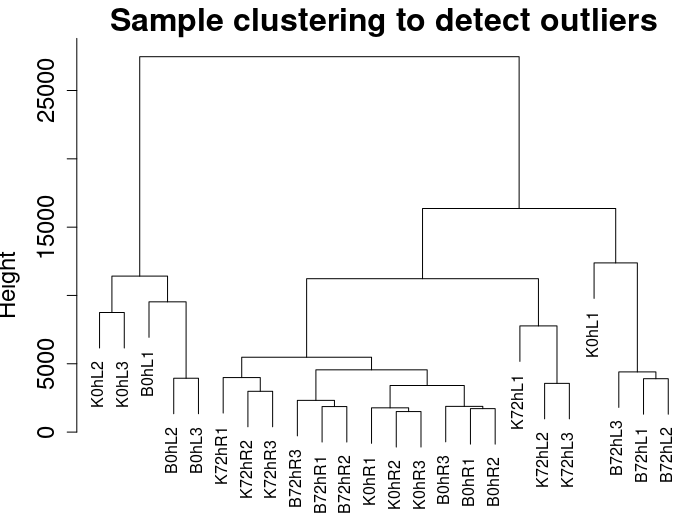

Supplement: Supplementary file 2 — Supplementary Material 2. [file 12870_2024_4932_MOESM2_ESM.zip › Supplementary figure/Supplementary figure 4.tiff]

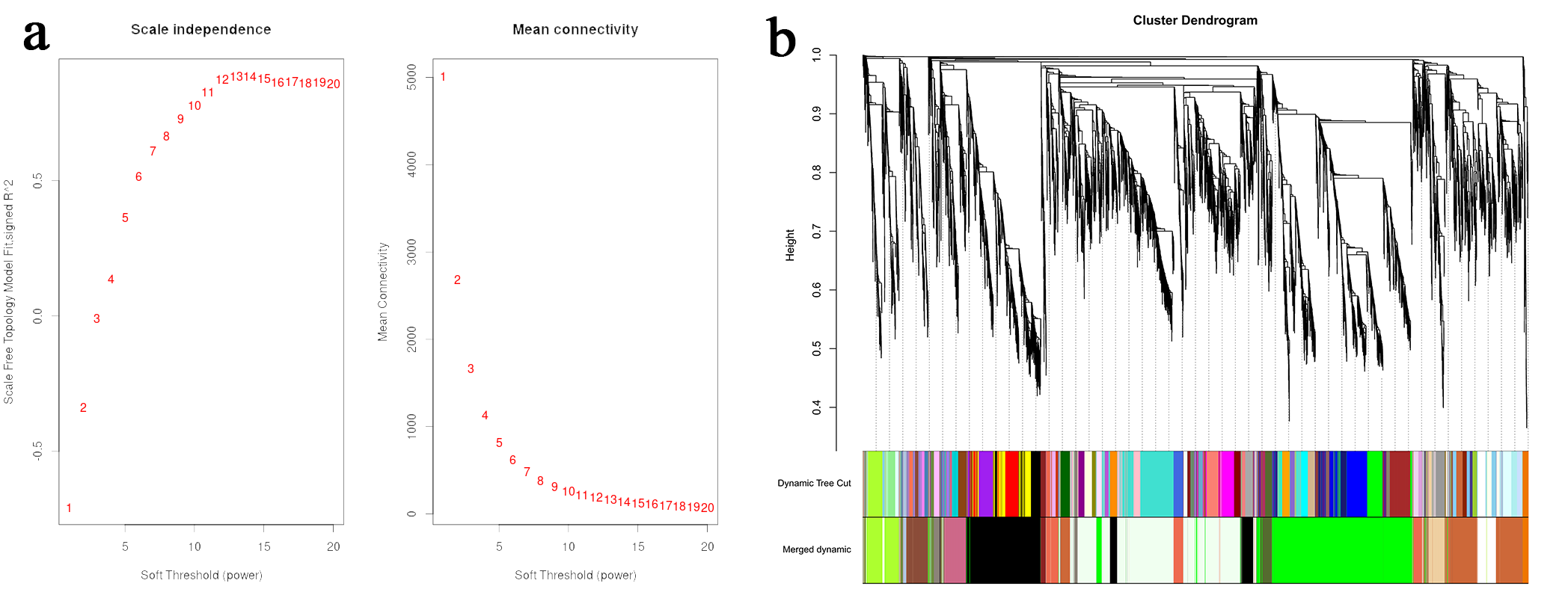

Supplement: Supplementary file 2 — Supplementary Material 2. [file 12870_2024_4932_MOESM2_ESM.zip › Supplementary figure/Supplementary figure 5.png]

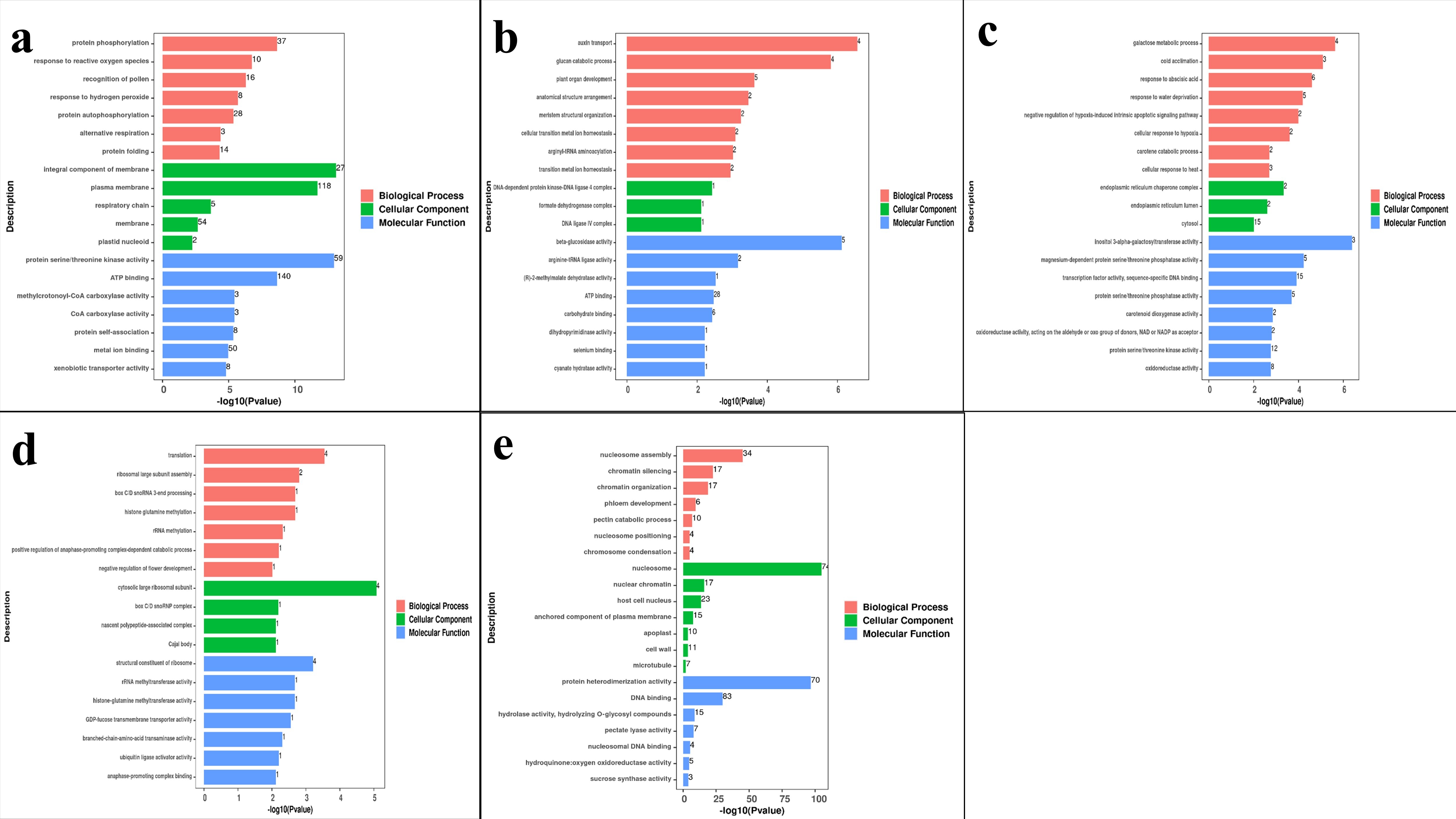

Supplement: Supplementary file 2 — Supplementary Material 2. [file 12870_2024_4932_MOESM2_ESM.zip › Supplementary figure/Supplementary figure 6.jpg]
